# A Metric for the Entropic Purpose of a System

**DOI:** 10.3390/e27020131

**Published:** 2025-01-26

**Authors:** Michael C. Parker, Chris Jeynes, Stuart D. Walker

**Affiliations:** 1 School of Computer Science and Electrical Engineering, University of Essex, Colchester CO4 3SQ, UK; 2 Independent Researcher, Tredegar NP22 4LP, UK

**Keywords:** entropy, teleonomy, information theory, origin of life, maximum entropy, drug discovery

## Abstract

Purpose in systems is considered to be beyond the purview of science since it is thought to be intrinsically personal. However, just as Claude Shannon was able to define an impersonal measure of information, so we formally define the (impersonal) ‘*entropic purpose*’ of an information system (using the theoretical apparatus of Quantitative Geometrical Thermodynamics) as the line integral of an *entropic* “purposive” Lagrangian defined in hyperbolic space across the complex temporal plane. We verify that this Lagrangian is well-formed: it has the appropriate variational (Euler-Lagrange) behaviour. We also discuss the teleological characteristics of such variational behaviour (featuring both thermodynamically reversible and irreversible temporal measures), so that a “*Principle of Least (entropic) Purpose*” can be adduced for any information-producing system. We show that *entropic purpose* is (approximately) identified with the *information created* by the system: an empirically measurable quantity. Exploiting the relationship between the *entropy production* of a system and its energy Hamiltonian, we also show how Landauer’s principle also applies to the *creation* of information; any purposive system that creates information will also dissipate energy. Finally, we discuss how ‘*entropic purpose*’ might be applied in artificial intelligence contexts (where degrees of system ‘aliveness’ need to be assessed), and in cybersecurity (where this metric for ‘*entropic purpose*’ might be exploited to help distinguish between people and bots).

## 1. Introduction

### 1.1. Entropic Purpose and Complex Time

This paper will show that a consistent technical definition may be made of a quantity that, because it is defined entirely in time, may be considered an attenuated form of *purpose*. We will call this quantity “*entropic purpose*”. It is necessary to emphasise that this narrow definition of “*entropic purpose*” is a scientific and impersonal quantity, entirely devoid of the philosophical properties of the *purposes* that dominate human affairs. It is true that there exist *teleological* implications of this scientific development which will be discussed, at length and separately, in an Appendix. But we note that the Principle of Least Action (which underpins much of the physical description of the universe and is elegantly described by Jennifer Coopersmith [1]) has teleological overtones (see Michael Stöltzner, 2003 [2]) yet is still unquestioningly considered to be rigorously scientific in nature.

What is additionally required for our new technical definition of *entropic purpose* is the systematic complexification of *time* (as well as space) that was demonstrated in 2023 (by Parker & Jeynes, **PJ23** [3]). This demonstration shows that complex time may coherently be thought to exist, a conclusion that has also been reached independently by others, as we will discuss.

### 1.2. Life and Artificial Intelligence

Raymond and Denis Noble [4] have asserted recently that, ”*Agency and purposeful action is a defining property of all living systems,*” (p. 47). This summarises the massive recent work in molecular biology and genetics that they discuss.

In the rapidly evolving landscape created by the transformative application of Artificial Intelligence (**AI**) techniques across a diverse set of domains, not only has the efficiency of complex systems been enhanced, but also the way has been paved for innovative engineering solutions to otherwise intractable problems (see for example Joksimovic et al. 2023 [5]). AI is therefore not just a powerful theoretical and practical tool in current engineering problems but is also now becoming a driving force shaping future technological advances.

One intriguing AI application investigates artificial life, seeking to emulate the performance of living systems and employing AI algorithms in an attempt to replicate biological processes (see for example Chan, 2019 [6]). The synergy of AI and artificial life investigations not only enriches our understanding of the complexities of life but also fuels the development of innovative technologies with the capability of profoundly changing the way we live our lives. The fusion of AI and life sciences (including molecular biology and biochemistry) could even offer new scientific insights into the origin of life studies (perhaps, see Yampolskiy 2017 [7]).

As society becomes increasingly reliant on interconnected digital communications, information, and computational systems, the importance of securing these networks against evolving threats is also becoming ever more critical. AI is emerging as a pivotal technology to help address the challenges posed by cybersecurity, and offering sophisticated solutions that adapt to the dynamic nature of cyber threats. The application of AI in security measures involves predictive analysis, anomaly detection, and intelligent response mechanisms, contributing to the creation of resilient and adaptive defence systems. In this context, one key aspect of identification and authentication is the ability to distinguish between real people (personalities) and the multiplicity of bots that attempt to impersonate and replicate the functions of actual people. Were it available, the ability to distinguish between animate and inanimate entities would be considered an important weapon in the arsenal of cybersecurity.

It is in this context that the work presented here offers an impersonal approach to measuring what we call the “*entropic purpose*” of a system. It is our expectation that the “*entropic purpose*” metric presented in this paper will be appropriate to biological and social applications since our ‘narrow’ definition allows it to be grounded in a mathematically rigorous and physical description appropriate for scientific application.

It is interesting to note that previous attempts to define the fundamental attributes of life have suggested various characteristics, including the following: cellular organisation; response to stimuli; growth; reproduction; metabolism; homeostasis; evolutionary adaptation; and heredity (see for example, Trifonov 2011 [8]). It is immediately obvious that *purpose* is currently (in general) not considered one of the attributes of life, perhaps because of the assumption that purpose cannot be considered a physically describable characteristic, being intrinsically *metaphysical* in nature and therefore not amenable to scientific methods of enquiry. However, everyone knows that living things have purposes (see [4] for example), and since living things are both real and physical then physics must be capable of describing these purposes.

### 1.3. Aristotelian Teleology

In this paper, we use “*metaphysical*” literally (*not* pejoratively, and *not* meaning “ontological”): that is, indicating “*the metanarrative of physics*” (with the broad Aristotelian meaning of *physics*: “pertaining to the natural world”). Jeynes et al. (2023) [9] have demonstrated the necessary existence of a metaphysical context of any physical discussion: although drawing attention to metaphysics is not currently considered scientifically proper, it must be acknowledged that every physical discussion is embedded in a metaphysical context.

“Purpose” is central to the Aristotelian physics that was overturned in the 17th century by Newtonian (mechanistic) physics: since then, any appeal to “purpose” has been regarded as scientifically illegitimate (with good reason!). However, *purposes* are manifestly a characteristic of life in general, and human activities in particular, so it seems perverse to deny their existence. It should be pointed out that many strands of research are pointing to a (partial) rehabilitation of Aristotelian ideas.

In the Graphical Abstract, Aristotle is looking at a Nautilus shell, obtained from the Indian Ocean. Surprisingly, we know he discussed this creature since it is documented in his “*Historia animalium*” (see von Lieven & Humar, 2008 [10]). Nautilus is of specific interest to us since it embodies the logarithmic spiral (on which see §2.4) and it is perhaps not surprising that the geometrical entropy description of such a (double) logarithmic spiral structure shares key mathematical features with the “purposive Lagrangian” (employed in this paper) that is associated with the creation of information. Although not the subject of this paper, there appears to be a deep and intriguing connection between the structural form of a living organism and its (entropic) purposeful function.

### 1.4. Shannon Information

When Claude Shannon developed his mathematical theory of information (1948 [11]), his fundamental underlying assumption was that such (essentially semantic) information *already* existed. Somewhat unexpectedly at the time (and his papers were immediately recognised as a critically important breakthrough), he was also able to show that such information could, in effect, be *de-personalised* and defined in a “scientific” (essentially syntactic) manner: the Shannon metric (known as “Shannon information” or “Shannon entropy”). In his studies, Shannon was interested in the engineering issues of the quantification of information and the conditions under which information can be faithfully transferred from one location in spacetime to another. In particular, Shannon defined the basis for a high-fidelity communications channel (consisting of a transmitter and a receiver, with a noisy channel in between), together with the conditions by which information at the transmitter end can be perfectly conveyed to the receiver end. It is clear that, ultimately, any such information must be *created* by a person who wishes to send it to another person able (and willing) to receive it: the information presupposes metaphysical purposes, but these purposes are irrelevant both to ours and to Shannon’s: his eponymous *information* was defined (impersonally) for the purposes of communications engineers, and our *entropic purpose* is defined (impersonally) for more general physical ends.

It is well known that information and noise have the same physical properties: both are intrinsically acausal (unpredictable and non-deterministic, see Hermann Haus, 2000 [12]). The key distinction between a string of noisy bits and a string of information bits is whether an algorithm exists (particularly at the receive end of a communications channel) to decode the received string. The existence of an algorithm (to first encode and then decode the information, making it robust against noise) bespeaks the existence of an intelligent (living) entity; but we are not here interested in intelligence per se, nor in its associated purposes.

### 1.5. Entropic Purpose

It is the aim of this paper to construct an impersonal (and therefore restricted) description of “*entropic purpose*”, in much the same way that a (restricted) concept of “information” was described in an impersonal way by Shannon. In effect, we will define an “*entropic purpose*” as being equivalent to the *creation* of such (Shannon) information, proving that any entity that creates (Shannon) information thereby exhibits *entropic purpose*.

The formalism presented here is based on the Quantitative Geometrical Thermodynamics of Parker and Jeynes (2019) [13]: the “prior art” is now very extensive and is summarised in §2, where the work itself is found in §3 with proofs of important relations given separately in Appendix A and Appendix B.

### 1.6. Teleonomy

*Teleonomy* is a neologism of Colin Pittendrigh of 1958, defined as “*evolved biological purposiveness*” by Corning et al. (2023) [14]. It is a circumlocution by the biologists to avoid using the term *Teleology*, which was thought to have too many Aristotelian overtones. Here, we intend to show how the teleological behaviour of systems (obvious in living systems) can be adequately represented in a valid *physical* account, thus making the term *teleonomy* redundant. These wider issues (not strictly *scientific* ones, although much science is discussed) are addressed more conveniently and at useful length in Appendix C.

### 1.7. Non-Technical Overview

This paper establishes the non-trivial result that a *purposive Lagrangian* exists. Roger Penrose, in his comprehensive work “The Road to Reality” (2004) [15], has a section entitled “*The magical Lagrangian formalism*” (his §20.1) in which he gives a beautiful account of the far-reaching power of this powerful mathematical framework. Of course, the difficulty is that constructing a Lagrangian for any given system is typically hard to do (the easy cases for ideal situations are rather rare). But here we give a “purposive Lagrangian” (as an example of our mathematical analysis) to act as a ‘template’ function. This may be adapted to the specific application in view: whether it be biological function, an emergent phenomenon due to the actions of multiple living organisms; the behaviour of a living entity as it goes about its purposeful activities (such as foraging for food); or indeed information creation due to some non-living (such as AI-based) system process.

However, we also show that the *entropic purpose* of a system (obtained from the Lagrangian) is essentially the same as the *information created* (not noise) by the system. That is, the *physical measure* of the *entropic purpose* of any system is simply the *new information created* by that system. Therefore, it is not actually necessary to construct the appropriate Lagrangian in order to calculate the *entropic purpose*. That is to say, if we can study a biological process (for example a foraging strategy) and calculate the information that is generated as the organism collects and processes sensory data (used to inform its decisions), then we will have also calculated the *entropic purpose* being exhibited by that organism. Thus, observation of the external phenomenological behaviour of the organism allows us to make a calculation of its *entropic purpose* without the need for knowing the underlying purposive Lagrangian that might be underpinning its actions: we can therefore acquire a quantitative evaluation in addition to the qualitative assessment of its ‘purposive behaviour’. It is true that we only treat the idealised case (in which noise is ignored or assumed zero) but we expect further work to show that the conclusion is generally valid (at least approximately).

The paper is quite long and technically difficult because the result that an impersonal *entropic purpose* can be mathematically defined is a radical surprise (going against deeply ingrained scientific assumptions), but rests on substantial previous mathematical works, while also requiring certain detailed technical criteria be established (Appendix A and Appendix B).

However, Appendix C considers a wide range of (mostly) recent work pointing to those same conclusions for which we now provide a solid physical basis. In particular, in Appendix C §C.1, we discuss how Samuel Butler (1835–1902) was convinced that “*life is matter that chooses*”: that is, the “*entropic purpose*” of an organism can be measured by its choices (effectively the *information* it generates). Similarly, in Appendix C §C.3, we discuss new observations of foraging birds (gannets and albatrosses) detailing how they *choose* their path to where the food is. Such choices are literally a matter of life and death.

The use of the word ‘path’ in the context of *entropic purpose* is intentional here since the key mathematical object employed in this paper is the line-integral of the purposive Lagrangian along a trajectory across the complex temporal plane. There is a phase associated with each incremental ‘infinitesimal purpose’ along the path of such a purposive trajectory, whose contributions to the overall integration are destructive or constructive according to the value of the phase at each point, such that the overall value of the integral is not a trivial result. Indeed, the purposive Lagrangian satisfies the Euler-Lagrange equation of variational calculus, so that there are an infinity of potential paths that a system could possibly follow, each associated with an overall *entropic purpose*. Yet, according to the *teleological* characteristic of the purposive Lagrangian, the actual path adopted by the system is the one that minimises the *entropic purpos*e and its associated information creation. We see here the *Principle of Least (entropic) Purpose*, such that a biological organism or purposive system exhibits an economy of behaviour, requiring the least amount of created information (that is, expending the least intellectual effort—an alternate rendering of the principle of Occam’s Razor) as it chooses to adopt a particular line of action or strategy in its attempt to achieve a desired objective (the *least entropic purpose*).

Interestingly, the purposive “line integral” is also an expression of a “non-local” constraint on the system, representing a “holistic” rather than a “reductionist” approach to finding how the system evolves (according to the variational calculus as exemplified by the Euler-Lagrange equation). Therefore, the purposive metric presented here implicitly describes (and is underpinned by) a highly complex and sophisticated mathematical mechanism with powerful analytical properties (although in a simple view it also merely represents the amount of information created by a system).

The reader may suspect that *entropic purpose* has something to do with *consciousness*, on the admittedly plausible grounds that our purposes are inextricably linked with our consciousness, and *information* is generated by conscious systems. There is enormous current interest in trying to express consciousness as the creation of information and its processing in complex neural systems. However, the present work sidesteps questions of consciousness by insisting that the purpose in view (*entropic purpose*) is entirely impersonal. These issues are addressed more fully in Appendix D.

However, by highlighting here the intuitively attractive idea that consciousness is closely related to the creation of information, we prove in this paper that as a physical phenomenon, information creation has a mathematical description that is both teleological (future-orientated) in nature and also obeys an underlying variational principle. That is to say, this variational principle (the principle of least entropic purpose) also implies the existence of deep symmetry relationships (and conservation laws, due to Noether’s theorem) within the broader concept of information creation. None of these critically important technical aspects associated with the creation of information have so far been discussed in the context of consciousness studies. Although this paper does not discuss these issues directly, we are now setting the scene in allowing consciousness research studies to be discussed more quantitatively within the context of these important mathematical principles, which are already known to underpin most (if not all) physical phenomena. It is in the context of these powerful principles that our paper breaks new ground in the literature of consciousness studies.

## 2. Technical Background

### 2.1. Holomorphism and the Lagrangian-Hamiltonian Representation

Parker and Walker (2004 [16]) have shown that a *holomorphic* function (a complex function analytic everywhere) *cannot* carry Shannon information, whereas a *meromorphic* function (piece-wise holomorphic) has poles with non-zero Cauchy residues and *can* carry information. Parker and Walker (2010) [17] have also considered the simplest case of a single pole (“point of non-analyticity”) in a restricted spacetime and shown that it obeys the Paley-Wiener [18] criterion for *causality*. Using geometric algebra methods [19], Parker and Jeynes (**PJ19** Appendix A [13]) formally generalise Parker and Walker’s 2010 treatment to Minkowski 4-space.

Quantitative Geometrical Thermodynamics (**QGT**: PJ19 [13]) systematically uses the well-known “*elegant unifying picture*” (Ch.20 in Penrose 2004 [15]) of the Lagrangian-Hamiltonian formulation, which Penrose explains in some detail saying that it leads to a “*mathematical structure of imposing splendour*”. In particular, QGT sets up an *entropic* Lagrangian-Hamiltonian system, proving that the required canonical equations of state are satisfied (see Equation (11) *passim* of PJ19 [13]).

Moreover, using this system, Parker and Jeynes (2023) [3] elegantly prove an equivalence between *energy* (represented by the Hamiltonian) and *entropy production*, using a systematic complexification of the formalism (including complexifying time itself), noting that, like energy, entropy production is a Noether-conserved quantity (proved by Parker and Jeynes, 2021 [20]). This is a startling development since it explicitly mixes up (or indeed, unifies) the reversible (where entropy production is identically zero) and the irreversible (with non-zero entropy production).

We will rely on this Hamiltonian-Lagrangian formulation, in particular setting up a “*purposive Lagrangian*” to enable the definition and discussion of “*entropic purpose*”. This is despite Roger Penrose saying, following a detailed technical discussion of the limitations of Lagrangians, “*I remain uneasy about relying on [Lagrangians] too strongly in our search for improved fundamental physical theories*” (*ibid*. §20.6, p. 491).

In classical mechanics, the Lagrangian represents a *balance* between potential and kinetic energy, and the *Least Action* is a minimisation of a temporal line integral along the Lagrangian. Such variational Principles are recognised as fundamental in physics: “*Least Action*” and “*Maximum Entropy*” have long been recognised, and “*Least Exertion*” was proved by Parker and Jeynes in 2019 [13], who also proved it to be equivalent to Jaynes’ “*Maximum Caliber*” (1980 [21], see Parker & Jeynes 2023 [22]). We should point out that although the *entropic* Lagrangian is in effect an *entropic* “balance” between the potential and kinetic entropies, this balance is not exact as in the case of classical mechanics. The situation is even more complicated for quantum mechanics: Paul Dirac addressed the issue in 1933 [23] and Richard Feynman famously took it up in his thesis [24] (see discussion by Hari Dass [25]). But note that Feynman explicitly says that his analysis is non-relativistic throughout, and Penrose points out (*ibid*. §20.6, p. 489) that “*strictly speaking*”, Lagrangian methods “*do not work*” for relativistic fields. However, the present QGT treatment is relativistic in principle [20].

### 2.2. Shannon Information and Info-Entropy

The impersonal definition of ‘*Entropic Purpose*’ that we will propose here relies on the mathematical and physical properties of the Shannon information, which is based on the mathematical functional object *ρ*ln*ρ*, where *ρ* is the probability distribution |Ψ|^2^ of a (complex) meromorphic wavefunction Ψ in Minkowski spacetime. The basic functional equations are given by Parker and Walker (2010, Equations (2) and (3) [17]) for the Shannon *entropy S* and the Shannon *information I*, and they give an integral over space *x*:

(1a) $$S\equiv \;k_{B}\int_{-\infty }^{\infty }\rho \left(x\right)\ln\rho \left(x\right)dx=k_{B}\ln ct\;$$

and a corresponding integral over time *t*:

(1b) $$I\equiv \;ik_{B}c\int_{-\infty }^{\infty }\rho \left(t\right)\ln\rho \left(t\right)dt=ik_{B}\ln x$$

where *ρ* is a meromorphic function with a simple pole at *x* + i*t* (i ≡ √−1 as usual), the Boltzmann constant *k_B_* is the quantum of entropy, and *c* is the speed of light. We generalise to natural logarithms and apply the proper metric [+++−] to the space/time co-ordinates (the Shannon information is imaginary compared to the Shannon entropy). Parker and Walker (2014) [26] also proved that a system in thermal equilibrium cannot produce Shannon information.

The logarithmic relations on the RHS of Equations (1a) and (1b) is a signature of *hyperbolic* spacetime which is also the theatre of QGT’s Hamiltonian-Lagrangian framework. Within the framework of QGT, Parker and Jeynes [13] also define the quantity *info-entropy f* of a system, proving that *f* is holomorphic:

(2) f=S+iI

where “holomorphic” means that the appropriate canonical spacetime-based Cauchy-Riemann relations are satisfied (see Annex to Appendix B of PJ19 [13]).

Considering a system where the geometric variation in 3D space occurs in a transverse plane described by the *x*_1_ and *x*_2_ co-ordinates along an axial co-ordinate *x*_3_, then the respective information and entropy of the system are simply the logarithms of its Euclidean co-ordinates [13], which can be written (somewhat informally):

(3a) $$S=\left(\ln x_{1}{\hat{\underline{x}}}_{1}-i\ln x_{2}{\hat{\underline{x}}}_{2}\right)\;k_{B}\;$$

(3b) $$I=i\left(\ln x_{2}{\hat{\underline{x}}}_{1}-i\ln x_{1}{\hat{\underline{x}}}_{2}\right)\;k_{B}$$

The info-entropy *f* of such a *x*_3_-axial system is given by:

(4) $$f=\ln\left(\frac{x_{1}}{x_{2}}\right)\left({\hat{\underline{x}}}_{1}+i{\hat{\underline{x}}}_{2}\right)\;k_{B}$$

where the logarithm argument is now formally correct, being explicitly dimensionless.

For a system comprising a double-helical geometry in 3D space, which is parametrically represented by the transverse co-ordinates *x*_1_ and *x*_2_ such that *x*_1_ = *R*cos(*κx*_3_) and *x*_2_ = *R*sin(*κx*_3_); the two helices are coupled by a pair of differential equations, where *x*_3_ represents the axial co-ordinate of the system and *κ ≡* 2π/λ is the wavenumber of the double-helix with λ its pitch and *R* its radius. Then we have:

(5a) $$\frac{\partial x_{1}}{\partial x_{3}}\equiv x_{1}^{\prime }=-\kappa x_{2}$$

(5b) $$\frac{\partial x_{2}}{\partial x_{3}}\equiv x_{2}^{\prime }=\kappa x_{1}$$

where the prime symbol indicates differentiation with respect to the spatial *x*_3_ axial co-ordinate. It is clear that using the coupled equations Equations (5a) and (5b), the info-entropy function *f* for such a double-helix eigenvector of QGT can be represented as:

$$f=k_{B}\ln\left(\frac{x_{2}^{\prime }}{\kappa x_{2}}\right)\left({\hat{\underline{x}}}_{1}+i{\hat{\underline{x}}}_{2}\right)\;\;\;\;\;\;\;\;\;\;\;\;\;\;\;\;\;\;\;$$

(6) $$=-k_{B}\ln\left(\frac{x_{1}^{\prime }}{\kappa x_{1}}\right)\left({\hat{\underline{x}}}_{1}+i{\hat{\underline{x}}}_{2}\right)+i\pi k_{B}$$

It is equally clear that the quantity $x_{n}^{\prime }/\kappa x_{n}$ (for *n* = 1,2) represents an important parameter for the double-helix eigenvector in QGT.

### 2.3. The QGT Equations of State

Quantitative Geometrical Thermodynamics (QGT) is a comprehensive and coherent description of the entropic behaviour of any system, entirely isomorphic to the well-known (kinematical) Hamiltonian and Lagrangian equations of classical mechanics. In constructing any such entropic equations of state, suitable “position” (*q*) and “momentum” (*p*) quantities need to be defined. In the application of QGT to creating a mathematical framework suitable for describing *entropic purpose*, these are defined for *n* ∈ {*t*,*τ*} in the complex temporal domain, where *Z*(≡*R*/*c*) is a temporal scaling factor analogous to the radius *R* of the double-helix in QGT (see Equation (9a) of [13]):

(7a) $$\mathrm{hyperbolic}\;\mathrm{time}:\;\;\;\;\;\;q_{n}\equiv Z\ln\left(\frac{n}{Z}\right)\;\;\;\;\;\;\;\;n\;\in \{t,\tau \}$$

(7b) $$\mathrm{entropic}\;\mathrm{momentum}:\;\;\;p_{n}\equiv {cm}_{S}/q_{n}^{\prime }={cm}_{S}q_{n}^{\prime *}\;\;\;\;n\;\in \{t,\tau \}$$

(7c) $$hyperbolic\;velocity\;q^{\prime }:\;\;\;\;\;\;\;\;\;\;\;q_{n}^{\prime }\equiv \frac{\partial q_{n}}{\partial T}=Z\frac{n^{\prime }}{n}\;\;\;\;n\;\in \{t,\tau \}$$

where *m_S_* ≡ i*κk_B_* is the entropic mass (Equation (9c) of [13]), *c* is the speed of light, and the prime symbols indicate differentiation with respect to the temporal parameter *T* (where *T* ≡ √(*t*^2^ + *τ*^2^), see formal treatment in §3 below and also Appendix A and Appendix B). The parameter *κ* is analogous to that described earlier, where *κ* = 2π/λ is the wavenumber with λ a pitch (see formal treatment in §3 below). The hyperbolic velocity *q*’ is dimensionless and *q*’* ≡ 1/*q*’: the ‘group’ velocity *q*’ is the inverse of the ‘phase’ velocity *q*’*, as normal (see Equation (9) of PJ19 [13], and §7 of Parker & Jeynes 2021 [27]), Note that Equations (7a)–(7c) means that equations of state for *entropic purpose* are defined in *hyperbolic* (not Euclidean) spacetime, and Equation (7c) shows how the hyperbolic velocity *q*^′^ relates to the associated Euclidean temporal derivative *n*^′^.

Considering the system entropy previously calculated using the Shannon entropy and indicated in Equation (1), it is clear that the hyperbolic time quantity *q* of Equation (7a) is functionally equivalent to the system Shannon entropy of Equation (1). That is to say, whereas we earlier merely noted that calculation of the Shannon entropy of a meromorphic point of non-analyticity is equivalent to the logarithm of the Euclidean spacetime co-ordinate, in QGT the hyperbolic position parameter *q* is functionally identical to the system Shannon information (except for the scaling factor *Z* and the quantum of entropy, *k_B_*). Thus, whereas the conventional mechanical equations of state are set within the theatre of Euclidean spacetime, the equations of state for *entropic purpose* and QGT are set within hyperbolic (logarithmic) space, and the transformation between the Euclidean and hyperbolic domains for a system can be seen to be effected by calculating the spatio-temporal *Shannon information* of the system (see Equations (1a) and (1b)).

The associated entropic momentum *p* as indicated in Equation (7b) is also intrinsically dependent upon the properties of hyperbolic time, but now via its temporal derivative *q*^′^; as determined by the axial co-ordinate *T*. In conventional mechanics, the momentum is given by the product of the inertial mass and the velocity; similarly, in QGT the entropic momentum is given by the product of the entropic mass *m_S_* (which includes the speed of light *c* for dimensionality reasons) and the (phase) hyperbolic velocity $q_{n\;}^{\prime *}$ ≡ 1/$q_{n\;}^{\prime }$.

We note the functional similarity of Equation (7c) to the quantity $x_{n}^{\prime }/\kappa x_{n}$ already identified in Equation (6). It is clear that to completely define the quantities seen in Equations (6) and (7c) only two parameters of a double-helix (a fundamental eigenvector of QGT) are required: the radius *R* (in this case the temporal parameter *Z* and the wavenumber *κ*). That is, these two parameters are sufficient to define the key equations of state that comprise the basis of QGT.

Thus, we emphasise that the Shannon information is completely intrinsic to (and permeates) the definitions and natures of both the entropic position *q* and the entropic momentum *p* in the hyperbolic spacetime of QGT. The corollary to this is that a universe exhibiting a hyperbolic geometry (such as ours, with its “hyperbolic overall geometry” as per Penrose’s assertion, §2.7 p.48, [15]) is also intrinsically informational in nature.

### 2.4. Irreversibility in QGT

**Figure 1:**
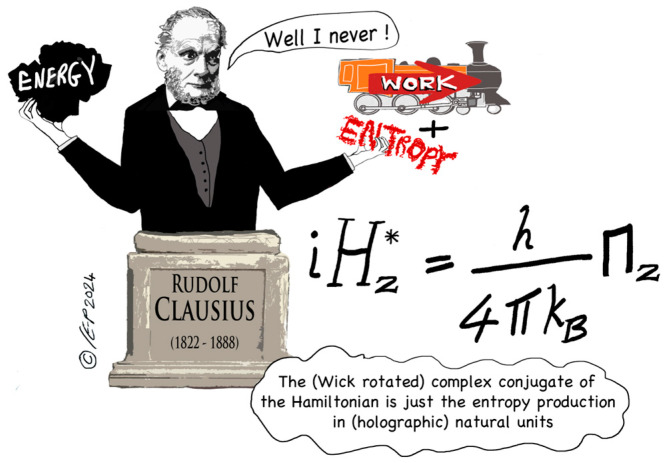
Graphical Abstract of “*Relating a System’s Hamiltonian to its Entropy Production using a Complex Time Approach*” (Parker and Jeynes 2023 [3]); *picture credit: Christine Evans-Pughe* (www.howandwhy.com).

Irreversible systems have positive (non-zero) entropy production. Parker and Jeynes (2019, [13]) have shown that the double logarithmic spiral (**DLS**, of which the double-helix is a special case) is a fundamental eigenfunction of the entropic Hamiltonian. But a DLS entails positive entropy production, as was shown by Parker and Jeynes’ (2021 [20]) QGT treatment of black holes (also proving that *entropy production* is a Noether-conserved quantity): this black hole treatment was confirmed subsequently in a more fundamental treatment (Parker and Jeynes 2023 [3]) which also established the essential physical equivalence of *energy* (as represented by the Hamiltonian) and *entropy production* (see Figure 1).

The double-helix has zero entropy production, which accounts for the stability of both DNA (PJ19 [13]) and Buckminsterfullerene (Parker and Jeynes 2020 [28]). In fact, a QGT analysis shows that the alpha particle (in its ground state) behaves as a *unitary entity* (than which exists nothing simpler: Parker et al. 2022 [29]). Where there is zero entropy production, there is no change—such cases are trivially *reversible*. But irreversible cases are where there is positive (non-zero) entropy production: all real processes involve change, and there is also a QGT treatment of the simplest such example—that of *beta-decay* (Parker and Jeynes 2023 [30]).

It is remarkable that QGT treats reversibility and irreversibility entirely commensurately: in fact, in a fully complexified treatment, the *Hamiltonian* (representing the system energy) and the *entropy production* are seen to be *equivalent*. This is a remarkable result: stated more precisely, the (Wick rotated) complex conjugate of the Hamiltonian is just the entropy production in (holographic) natural units ([3]; see Figure 1). But this result is only obtained in a fully complexified system, that is, where *time* is also complexified. Ivo Dinov’s Michigan group refers to the resulting 5-D spacetime as “*spacekime*”, where “kime” refers to “complex time” (see for example Wang et al. [31]).

The world is irreversible. Therefore, the fundamental equations of physics cannot be reversible. Consequently, QGT takes the (irreversible) *Second Law of Thermodynamics* as axiomatic. *Purpose* is an intrinsically irreversible concept, a truncated form of which (“*entropic purpose*”) we will express here using the (QGT) apparatus of complex time.

### 2.5. Other Comments

Information and noise are physically indistinguishable in a Shannon communication channel: this is why “cryptographically secure pseudo-random number generators” are of such importance in establishing secure communications. Formally, “entropy” is added to the information at the transmit end, and then subtracted at the receive end to retrieve the information. Therefore, information production (the rate of information creation) and entropy production (the rate of increase in entropy) are also closely related.

The ”*fundamental equations of physics*” are usually considered to be those of Quantum Mechanics (QM) and General Relativity (GR), which have both been demonstrated correct by multiple very high-precision experiments. The trouble is that QM is apparently in “*fundamental conflict*” with GR (Penrose [15] §30.11): Roger Penrose is convinced that QM “*has no credible ontology, so that it* **must** *be seriously modified for the physics of the world to make sense*” (*ibid*. §30.13 p. 860, emphasis original). Many physicists do not take Penrose’s view; nevertheless, there is still no consensus on a theory of quantum gravity. We take the view that “*fundamental equations*” should treat reversibility and irreversibility commensurately (which neither QM nor GR do) on the grounds that irreversibility is a phenomenon ubiquitously observed.

In fact, there are no real systems that are reversible! QM has looked for the “*fundamental particles*”, but the smaller they are, the higher the energy needed for the relevant experiments—at such high energies the assumption of reversibility is an exceedingly good approximation. But QGT has shown that there is another approach to the fundamental: Parker et al. 2022 [29] have shown that it is reasonable to consider the alpha particle (in its ground state) as a *unitary entity* (than which exists nothing simpler): that is, at its unitary length scale the alpha as such should also be considered “fundamental” (since there exists nothing simpler)—even though we know it is composed of two protons and two neutrons (when considered from the reference frame of a smaller length scale); a system of four nucleons which is complicated compared to the unitary entity. Hyperbolic space emphasises the *relativity of scale* (see Auffray & Nottale’s useful review 2008) [32]. Of course, if the alpha is excited (not in its ground state), it behaves as a *system*, not as a *unitary entity* (which has no parts).

## 3. A Formal Expression for a System with a Non-Zero Entropic Purpose

### 3.1. Overview

We will take an engineering approach, idealising the problem to facilitate an analytic solution. Centrally, we use the result of **PJ23** ([3], see Figure 1) that *time* must be represented as a complex number to adequately express the Second Law (in turn allowing the expression of the irreversibility ubiquitously observed for all real systems), finding that it is possible to construct a well-formed *purposive* Lagrangian in the complex temporal plane: that is, without a spatial component. We call this Lagrangian “purposive” on the grounds that *purpose* is a temporal phenomenon without a spatial component. Of course, we cannot set up a physical system to adequately represent the full scope of purposes (including mental phenomena): our formalism is necessarily restricted to the purely physical.

To underline this, we, therefore, refer here to “*entropic* purpose”. We acknowledge that, since the Newtonian revolution of the 17th century, assigning “purpose” (of any sort) to things is regarded as illegitimate in physics. However, the introduction of complex time means that the classical Lagrangian-Hamiltonian apparatus can be extended to irreversible cases, including ones that can be interpreted as *purposive*.

We will define the Entropic Purpose *P* [J/K] as an appropriate line integral along the relevant (“purposive”) Lagrangian *L_P_*, in the same way that the action (or the “exertion”, see Equation (12) of PJ19 [13]) are line integrals on the appropriate Lagrangians. We will also show that just as there is a *Principle of Least Action* and a *Principle of Least Exertion* [13], there is also a *Principle of Least (entropic) Purpose* (see Appendix A). Note parenthetically that Parker and Jeynes (2023 [22]) have shown that the “exertion” is proportional to what Edwin Jaynes called “caliber” (1980 [21]), and Pressé et al. [33] have emphasised the general nature of the variational Principle of “*Maximum Caliber*”.

**Figure 2:**
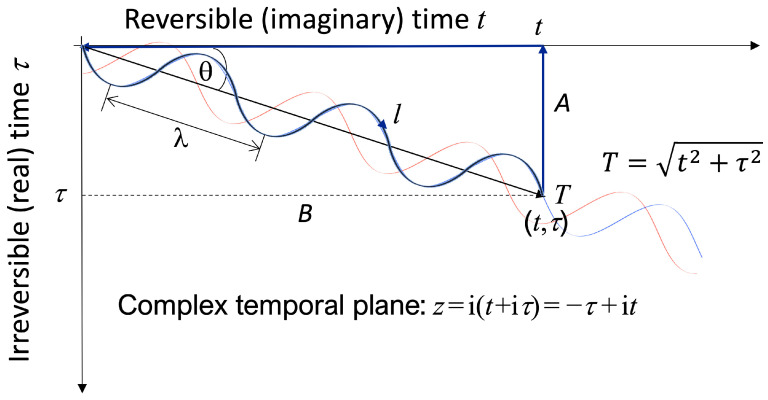
Description of a holomorphic trajectory *l* (represented by the red-blue pair of lines) across the complex *z* temporal plane. Note that because *z* = −τ + i*t,* the real-time (*tau*) axis is inverted, implying an intrinsic handedness (chirality) to the complex time plane.

Purpose involves time, and purposive systems must follow some sort of trajectory through time. The treatment of information in QGT is predicated on the existence of meromorphic functions which are piece-wise holomorphic, with the (particle-like) poles of the function behaving like pieces of information. Figure 2 sketches an example trajectory *l* through complex time of one such pole. This trajectory may readily be constructed holomorphic: see Appendix A Equation (A1a).

As well as defining the holomorphic trajectory *l* (see Equation (A1a) of Appendix A) also shows that the associated “purposive Lagrangian” (see §§3B,3C) is well-formed; that is, it satisfies the appropriate (entropic) Euler-Lagrange equation in which the conjugate variables {*p*,*q*} of Equation (7) are defined in a hyperbolic complex time {*t*,*τ*} (QGT is always defined in an information space that is necessarily hyperbolic). Entropic position *q* and entropic momentum *p* = *cm_S_q*’* (with entropic mass *m_S_* ≡ i*κk*_B_) are all defined as previously (see Equations (7a)–(7c) *passim*), except that the prime now indicates partial differentiation by the temporal parameter *T* shown in Figure 2 (*q*’ ≡ ∂*q*/∂*T*: but note also that *q*’* ≡ 1/*q*’ as before).

We can calculate the entropic purpose directly (§3.4) by interpreting the line integral along the “purposive Lagrangian” across the complex temporal plane as the *entropic purpose* of the system. We can also complexify the Lagrangian to make it analytic (see Appendix B) so as to conveniently exploit the mathematical apparatus of complex algebra and calculate a closed path integral (with zero residue).

Finally, we calculate the *Information* created (§3.5). Here, we consider an idealised system unaffected by noise: that is, we assume that all the entropy produced by the purposive system is informative. The entropy produced by the system (in this idealisation, all in the form of information) is calculated as usual by a line integral across the *purposive Hamiltonian*, which is obtained from the Lagrangian as is conventional by the appropriate Legendre transformation.

The aim is to obtain a formal expression of *entropic purpose*, see Equations (19a,b); together with the corresponding *Shannon information,* see Equation (25). We discuss the metaphysics associated with *entropic purpose* (that is, this truncated treatment of *purpose*) in light of our new physical results in Appendix C.

### 3.2. The Purposive Lagrangian L_P_

A new “*Purposive Lagrangian*” *L_P_* is defined in the complex temporal plane (thus having no spatial component, just as *purpose* is purely temporal), using the Lagrangian for the double-logarithmic spiral (shown in PJ19 [13] to be a fundamental eigenfunction of the entropic Hamiltonian) as a template: see Equation (A10a,b) of Appendix A:

(8) $$L_{P}=cm_{S}\left(\ln q_{t}^{\prime }+\ln q_{\tau }^{\prime }\right)+2cm_{S}\left[\Lambda T-\ln\left(1-\Lambda T\right)\right]+cm_{S}\ln K_{t}K_{\tau }\;$$

where as usual *c* is the speed of light, *k_B_* is the Boltzmann constant, and *κ* ≡ 2π/*λ* is a wavenumber corresponding to the parameter λ representing the length scale of the system trajectory. *K_n_* (*n* ∈ {*t*,*τ*}) are entropic constants. Here, *m_S_* is the “entropic mass” which is a constant of the purposive system that scales with *κ* (*m_S_* ≡ *iκk_B_*: see Equation (9c) of [13] *passim*). Time is expressed as a complex number with real (*τ*) and imaginary (*t*) parts expressing respectively the irreversible and reversible behaviours of the system.

The parameter Λ was used previously to represent the logarithmically varying radius of the double logarithmic spiral (in a hyperbolic 4-D Minkowski space, see Equations (20) and (D.6) of PJ19 [13]). Here, it is used in a way that is formally similar but now in the (hyperbolic) 2-D space given by the complex time plane. Here we interpret it as characterising the “*entropic purpose*” of the system, such that Λ ≥ 0, and when Λ = 0 the system has zero *entropic purpose*.

In addition, the complex quantity *T*, which in Figure 2 represents a temporal point on the holomorphic trajectory across the complex time plane, is a critically important aspect of the geometry. Its main physical interpretation, as a *scalar* quantity, is that it represents the least required time (apparently known in advance) to produce a given amount of information by a system. Thus, a system creating a certain amount of information will require a system-determined *minimum* period of time *T* (calculable in advance) to produce that information.

The time *T* can also be understood to be the *empirically measurable* time that elapses over the course of the information-producing process. That is to say, the measured time *T* (which must be monotonically increasing according to the 2nd Law) is a (vectorial) function of the (mutually orthogonal) reversible and irreversible times. Any information-producing process proceeds along a holomorphic trajectory *l* in the temporal plane, but it is the monotonically increasing resultant time *T* that is measured, and which also represents the key variational parameter of the Lagrangian calculus. Just as the *Principle of Least Time* (or more generally, the *Principle of Least Action*) offers a minimum time interpretation for any physical phenomenon, so the time *T* can also be (loosely) understood to represent the minimum time to produce a given amount of information according to a *Principle of Least (entropic) Purpose* (see Appendix A).

The key canonical relationships (of the Euler-Lagrange variational equation involving Hamiltonian and Lagrangian quantities) are defined using QGT in hyperbolic spacetime, as discussed above and as appropriate for an analysis based on the Shannon information. The Euclidean complex temporal plane is given by *z* = i(*t* + i*τ*) (see Equation (3) of [3]) as indicated in Figure 2. The associated hyperbolic complex time is then given by *q_n_*/*Z* = ln(*n*/*Z*): *n* ∈ {*t*,τ} (see Equation (7a) above) where in this context *Z* is the holographic temporal scaling factor (akin to a ‘temporal radius’) of the system (the equivalent holographic spatial radius *R* is discussed above, see Equation (5a,b); and *q*′ is the derivative of hyperbolic time with respect to *T*, given by *q*′*_n_* ≡ ∂*q_n_*/∂*T*: *n* ∈ {*t*,τ} (see Equation (7b): combined with the entropic mass *m_S,_* these are effectively the entropic momenta). Finally, the quantity Λ in Equation (8) is the parameter that determines the strength of *entropic purpose*: Λ = 0 for a system with zero *entropic purpos*e.

The first term on the RHS of Equation (8) can be considered to be a ‘kinetic’ term since it is composed of temporal derivatives, whereas the second term on the RHS can be considered as the ‘potential’ term of the Lagrangian since it is determined by non-derivative (i.e., “field”) considerations. The final term on the RHS is related to the granularity or scale of the physical system that is exhibiting *entropic purpose* (again noting that the *entropic purpose* in a hyperbolic representation requires the frame of scale to be explicitly referenced, see Auffray and Nottale [32]), and is a constant, akin to a constant of integration or an offset term (as is generally present in considerations of entropy).

Appendix A shows the purposive Lagrangian of Equation (8) to be valid by demonstrating that it satisfies the Euler-Lagrange equation describing the appropriate variational principle, which we will call the *Principle of Least (entropic) Purpose*:

(9) $$\frac{d}{dT}\frac{\partial L_{P}}{\partial q_{n}^{\prime }}-\frac{\partial L_{P}}{\partial q_{n}}=0$$

for both temporal dimensions *n* ∈ {*t*,τ}. That is, across the complex temporal plane *z*, given by *z* = −τ + i*t*, the *Principle of Least (entropic) Purpose* is obeyed by an information-producing system exhibiting the Lagrangian of Equation (8), such that the system adopts a holomorphic trajectory *l* across the complex temporal plane. Therefore, the *entropic purpose P* of the system is given by the line integral of the purposive Lagrangian along the trajectory across the complex time plane as the information-creating process evolves:

(10) $$P=\int_{l}L_{P}\left(q,q^{\prime },T\right)dz$$

where d*z* represents the infinitesimal time increment along the trajectory as it traverses the complex temporal plane. Since the trajectory *l* obeys the variational calculus of the Euler-Lagrange equation of Equation (9), it also indisputably has properties that appear to be teleological. That is to say, before the process starts, the minimised *entropic purpose P* of the system already determines what the (empirically-measurable) temporal duration *T* of the process will be.

### 3.3. Calculating L_P_

We now calculate the *entropic purpose P* of a system that exhibits the purposive Lagrangian of Equation (8). The hyperbolic derivatives in the complex temporal plane are given by:

(11a) $$\frac{\partial q_{t}}{\partial T}\equiv q_{t}^{\prime }=i\kappa Z\sin\theta \left(1-\Lambda T\right)$$

(11b) $$\frac{\partial q_{\tau }}{\partial T}\equiv q_{\tau }^{\prime }=i\kappa Z\cos\theta \left(1-\Lambda T\right)$$

where the angle *θ*, seen in Figure 2, is the local orientation of the trajectory across the temporal plane; see Appendix A, Equations (A6a) and (A6b). The characteristic holographic temporal parameter (analogous to the geometric radius) of the system is given by *Z*_0_, assumed constant. This allows us to simplify the purposive Lagrangian of Equation (8) (see Equation (A10b) in Appendix A):

(12) $$L_{P}=cm_{S}\ln\left(\sin2\theta \right)+cm_{S}\ln\left(-\kappa^{2}Z_{0}^{2}/2\right)+cm_{S}\ln K_{t}K_{\tau }$$

Here we see that the purposive Lagrangian is composed of a variable component (depending on the angle *θ*) and an essentially overall constant component (the two final terms in Equation (12) on the RHS). In a dimensionally consistent expression, the angle *θ* may be given by:

(13) $$\theta =\tan^{-1}\left(\frac{\tau }{t}\right)$$

which exhibits plausible behaviour as the value of Λ varies. In particular, the angle *θ* may reasonably be supposed proportional to the information production parameter Λ, see Equation (20), and we therefore employ the wavenumber *κ* as the dimensionally equivalent system parameter to normalise Λ. For example, when Λ = 0 then the angle *θ* = 0, and the trajectory of the system simply proceeds along the reversible *t*-axis: no information is being produced and zero thermodynamic irreversibility (as represented by the temporal parameter *τ*) is present. However, for finite Λ > 0 then the angle is also *θ* > 0, and the irreversible temporal parameter *τ* is also finite. We need only consider the varying part of Equation (12), the purposive Lagrangian that explicitly depends on the information production, given by:

(14a) $$L_{P}=cm_{S}\ln\left(\sin2\theta \right)$$

However, in the subsequent analysis, we consider the analytically-continued (complexified according to the handedness of the *z = −τ +* i*t* plane) version of the purposive Lagrangian, which is given by (see Equation (A15b) of Appendix B):

(14b) $$L_{P}=cm_{S}\ln\left(\sin2\theta +i\cos2\theta \right)$$

This complexified purposive Lagrangian can then be immediately simplified, using the Euler identity sin2*θ* + icos2*θ* = iexp(−i2*θ*), to:

(14c) $$L_{P}=-i2cm_{S}\theta$$

which we use in the subsequent analysis, and where we have ignored the constant term associated with ln(i) in the purposive Lagrangian of Equation (14c) (and also other constant terms in Equation (12) on the RHS), since constant aspects to any Lagrangian (or Hamiltonian) play no part in the system dynamics as described by the relevant variational calculus and canonical differential equations. That the purposive Lagrangian is analytic in the complex temporal *z*-plane (see Appendix B) is also helpful.

### 3.4. Calculating the Entropic Purpose P

With reference to Figure 2 and given there are no poles or points of non-analyticity enclosed within the closed contour described across the complex temporal plane; using the fundamental results of complex calculus, we can see that a closed contour (line) integral of the purposive Lagrangian must equal zero, tracing from the origin along the trajectory *l* to the temporal point *T* at (i*t*,*τ*), and then ascending vertically upwards (parallel to the *τ*-axis) along the line *A*, and then backwards (along the *t*-axis) on the line *B* back to the origin:

(15) $$\oint L_{P}\left(q,q^{\prime },T\right)dz=\int_{l}L_{P}dz-\int_{A}L_{P}d\tau +i\int_{B}L_{P}dt=0$$

Of course, this requires the purposive Lagrangian to represent an analytic function in the temporal complex *z*-plane. Our previous work (PJ23 [3]) allows us to assume that the Cauchy-Riemann conditions hold as appropriate for the handedness of the *z*-plane (see Appendix B): $\partial L_{P,r}/\partial t=-\partial L_{P,i}/\partial \tau$ and $\partial L_{P,i}/\partial t=\partial L_{P,r}/\partial \tau$, where the complexified purposive Lagrangian is composed of two real functions: $L_{P}=L_{P,r}+iL_{P,i}$. That is to say, just as QGT’s entropic Hamiltonian of [3] is complex, whose real (dissipative and entropic) and imaginary (reversible and energetic) components are Hilbert transforms of each other, the QGT entropic Lagrangian (the simple Legendre transformation of the entropic Hamiltonian) is also complex and analytic. Given that the *purposive* Lagrangian *L_P_* is based on equivalent QGT quantities (although now defined in the complex temporal plane), Appendix B shows that *L_P_* is also analytic.

Fortunately, the two line-integrals *A* and *B* can be calculated analytically, so that the *entropic purpose* integral of Equation (10) can be evaluated. We consider the two integrals along the paths *A* and *B* in turn.

*Line integral A* is calculated using Equations (14c) and (13):

(16) $$-\int_{A}L_{P}d\tau =-\int_{\tau }^{0}-i2cm_{S}\theta d\tau =i2cm_{S}\int_{\tau }^{0}\tan^{-1}\left(\frac{\tau }{t}\right)d\tau =i2cm_{S}{\left[\tau \tan^{-1}\left(\frac{\tau }{t}\right)-\frac{t}{2}\ln\left(t^{2}+\tau^{2}\right)\right]}_{\tau }^{0}\;=-i2cm_{S}\left(\tau \theta +t\ln t/T\right)\;\;$$

*Line integral B* is also calculated using Equations (14c) and (13):

(17) $$i\int_{B}L_{P}dt=i\int_{t}^{0}-i2cm_{S}\theta dt=2cm_{S}\int_{t}^{0}\tan^{-1}\left(\frac{\tau }{t}\right)dt=2cm_{S}\int_{t}^{0}\frac{\pi }{2}-\tan^{-1}\left(\frac{t}{\tau }\right)dt\;=2cm_{S}{\left[\frac{\pi }{2}t-t\tan^{-1}\left(\frac{t}{\tau }\right)+\frac{\tau }{2}\ln\left(t^{2}+\tau^{2}\right)\right]}_{t}^{0}=2cm_{S}\left(\tau \ln\frac{\tau }{T}-t\theta \right)$$

Thus:

(18) $$-P=-\int_{A}L_{P}d\tau +i\int_{B}L_{P}dt=2cm_{S}\left\{\left(\tau \ln\frac{\tau }{T}-t\theta \right)-i\left(\tau \theta +t\ln\frac{t}{T}\right)\right\}$$

and therefore, the *entropic purpose P* has real and imaginary components given by:

(19a) $$P_{r}=2cm_{S}\left(t\theta -\tau \ln\frac{\tau }{T}\right)\;\;\;\;\;\;$$

(19b) $$P_{i}=2cm_{S}\left(\tau \theta +t\ln\frac{t}{T}\right)$$

It is clear from Equations (19a,19b) that the dimensionality of *entropic purpose* is given by entropy [J/K] and (given the definition of the entropic mass *m_S_*) that it is quantised by the Boltzmann constant *k_B_*. In addition, since the complexification of the (real) purposive Lagrangian of Equation (14a) led to a complex-valued *entropic purpose*, it is clear that it is the real part of the *entropic purpose*, Equation (19a), that we are interested in.

That is to say, we need only consider the real component, *P_r_* (Equation (19a)) which is simply the result of the line integral *B* along the reversible time axis (but not independent of the line integral *A* along the irreversible time axis due to the Hilbert transform relationship between the real and imaginary components of the complexified purposive Lagrangian). This is because for a system with zero information production (*θ* = 0 and *τ* = 0), then *P_r_* = 0. However, as the information production increases, such that *θ* > 0 and *τ* > 0, then *P_r_* > 0, as expected for a system that is creating information. In addition, it is clear that the *entropic purpose* increases over time as more information is created by the system.

As a first-order heuristic approximation, and because when no information is being produced, both *θ* and Λ are zero, we can conjecture that the angle *θ* is related to the information production parameter Λ (dimensioned correctly with the factor *κ*) by:

(20) $$\theta =\frac{\Lambda }{\kappa }$$

### 3.5. Calculating the Information

Having calculated the *entropic purpose* of an information-producing system, the next question that immediately comes to mind is, how much information is this *entropic purpose* associated with? Whereas in QGT the exertion (as associated with the Principle of Least Exertion) is found by calculating the line integral of the entropic Lagrangian, the entropy associated with the system (assumed to be all in the form of information in this idealised system) is found by calculating the line integral of the entropic Hamiltonian over the same trajectory. Thus, whereas the *entropic purpose* (with the dimensionality of entropy, [J/K]) is calculated by the temporal line integral of the purposive Lagrangian, the associated increase in Shannon information (itself also an entropic quantity with the same dimensionality, [J/K]) corresponds to the line integral of the purposive Hamiltonian over the same trajectory across the complex temporal plane.

The purposive Hamiltonian *H_P_* and purposive Lagrangian *L_P_* are related to each other by the Legendre transformation:

(21) $$H_{P}=\sum_{n=t,\tau }q_{n}^{\prime }p_{n}-L_{P}$$

The identity $q_{n}^{\prime }p_{n}={cm}_{S}$ (see Equation (7b) above, and Equation (9b) of PJ19 [13]) shows that the first term on the RHS of Equation (21) does not vary over time. Then it is clear from Equation (14a) that the varying part (that depends on the angle *θ* as the temporal trajectory crosses the complex time plane) of the purposive Hamiltonian is simply given by the purposive Lagrangian:

(22) $$H_{P}=-cm_{S}\ln\left(\sin2\theta \right)$$

Any constant aspect to the purposive Hamiltonian simply contributes to the (constant) offset associated with the information entropy and can be ignored, since we are only interested in the change in information (i.e., the information production). Therefore we can deploy the same complex analysis as for the calculation of the *entropic purpose P* above, and we complexify the purposive Hamiltonian of Equation (22) in the same way as Equations (14a)–(14c):

(23) $$H_{P}=i2cm_{S}\theta$$

In the same way that we found that the real part of the *entropic purpose* was simply the line integral along the reversible time axis, we calculate the information *I* along the same line integral. Note, that from an info-entropy perspective, where information and entropy are in quadrature to each other (that is, using the language of geometrical algebra, their basis vectors are Hodge duals of each other [13]) and the line integral of an entropic Hamiltonian gives the entropy of a system [3], so we take the complex-conjugate of the purposive Hamiltonian of Equation (23) and multiply by the pseudoscalar i so as to yield the appropriate information term *I* arising from the integration:

(24) $$I=\int_{0}^{t}iH_{P}^{*}dt$$

and realise that we obtain exactly the same result as for *P*_r_ see Equation (19a):

(25) $$I=i\int_{0}^{t}-i2cm_{S}\theta dt=2cm_{S}\int_{0}^{t}\tan^{-1}\left(\frac{\tau }{t}\right)dt=2cm_{S}\int_{0}^{t}\frac{\pi }{2}-\tan^{-1}\left(\frac{t}{\tau }\right)dt\;=2cm_{S}{\left[\frac{\pi }{2}t-t\tan^{-1}\left(\frac{t}{\tau }\right)+\frac{\tau }{2}\ln\left(t^{2}+\tau^{2}\right)\right]}_{0}^{t}\;=2cm_{S}\left(t\theta -\tau \ln\frac{\tau }{T}\right)$$

It is clear that the created information *I* of Equation (25) behaves as expected: in conjunction with Equation (20), it is zero for Λ = 0, and increases with Λ just as the *entropic purpose P* increases with Λ. We also see that the *entropic purpose* and the *created information* are identical. That is to say, they imply each other: the creation of information implies *entropic purpose*, and vice-versa.

Note that *entropy production* is a conserved quantity, just like energy. One way of understanding this is to remember that the energy Hamiltonian *H* and the entropy production Π are mutually complex conjugates (Equations (23) of PJ23 [3]) and since the Hamiltonian is Noether-conserved, so is the entropy production. But the entropy production of a purposive system is also given by the sum of its information production and the associated ’noise production’ (which all have the same units). The sum of the information production and noise production is therefore conserved, but the interplay between the information and noise means that the productions of each are *not* individually conserved at each instant in time. This is similar to the process of transformations over time between kinetic energy and potential energy in a dynamic system, where the total energy is a constant (conserved) but at each instant in time the amount of KE and PE in the system is variable. Similarly, we can speak of the generation (creation) of information as part of a dynamic process where the overall entropy production of the system is conserved (a constant), but the relative allocations to information production and noise generation can vary with time.

Given that the time to create the information is *T*, then using Equations (A2a) and (20), the information production Π is obtained from Equation (25):

(26) $$\Pi =\frac{\partial I}{\partial T}=2cm_{S}\frac{\tau }{T}=2cm_{S}\sin\theta =2cm_{S}\sin\frac{\Lambda }{\kappa }\;\;$$

From Equation (23c) of PJ23 [3], the associated energy Hamiltonian, using *m_S_* ≡ i*κk_B_*, is then:

(27) $$H=i\frac{h}{4\pi k_{B}}\Pi^{*}=hf\sin\theta =hf\sin\frac{\Lambda }{\kappa }$$

where $\Pi^{*}$ is the complex conjugate of Π and *c* = *fλ* as usual. Note that for an angle *θ* = 90° across the temporal plane, the energy *H* associated with the information production is therefore simply that of a photon of frequency *f*.

Equation (27) indicates that any system exhibiting *entropic purpose* (that is, creating Shannon information) will also dissipate energy. This is an interesting extension to Landauer’s principle [34,35] (see also [17] and a recent review [36]), which conventionally states that the deletion (erasure) of information requires energy; that is, it must be a dissipative process. In earlier work, Parker and Walker [16] already showed that the *transfer* of information is also dissipative. However, now we can also see that the *creation* of information is equally dissipative. Thus, the physical processing of any information (be it its creation, copying, transfer, or erasure) is always accompanied by the dissipation of energy.

Note that although the sign of the information production can change according to the physical process (so that the creation of information is associated with a positive sign for ∂*I*/∂*T*, whereas its destruction or erasure will have a negative sign), yet because the information production is the Wick-rotated complex-conjugate of the energy Hamiltonian, the sign of the energy change is ambiguous, as indeed, is the sign of the associated temporal change. However, given that the 2nd Law is fundamental, the energy change must always manifest itself as a dissipation event, accompanied by an overall increase in entropy.

### 3.6. Communication Systems Power Requirements

From an engineering perspective, there is always an interest in minimising the energy dissipation or powering requirements for any communications system. The quotient of Equations (26) and (27) indicates that the energy required for a given information production is:

(28) $$\frac{H}{i\Pi^{*}}=\frac{\hbar }{2k_{B}}\;\;\;\;[\mathrm{Ks}]$$

The dimensionality of Equation (28) needs a comment, in that the information production rate [s^−1^] is quantised by the Boltzmann constant [J/K], such that the information production Π has overall dimensionality [J/Ks], whereas that of the energy Hamiltonian *H* is simply [J]. Thus Equation (28) offers a theoretical minimum energy dissipated per rate of created information; however, the energy is expressed in Kelvin-seconds. In order to express it in a more familiar form, Landauer’s principle specifies the minimum energy dissipation per (erased) bit:

(29) $$k_{B}T\ln2\;\;\;\;[J/b]$$

That is to say, we multiply Equation (28) by the thermodynamic (entropic) unit value for a bit of information, *k_B_*ln2, such that the minimum energy for a given information creation rate is:

(30) $$\frac{\hbar }{2k_{B}}\;k_{B}\ln2=\hbar \frac{\ln2}{2}\;\;\;\;[J/b/s=\mathrm{Js}/b]\;$$

It is interesting that the minimum energy for a given information production depends on the Planck constant, rather than the Boltzmann constant; particularly since ℏ (1.055 × 10^−34^ Js) is numerically considerably smaller than *k_B_*, (1.381 × 10^−23^ J/K) albeit they are different physical quantities. However, only temperatures in the nanokelvin range (for example) that are more associated with black holes or very advanced optical cooling techniques would yield energy values equivalent to 1 b/s information creation rate, or at room temperature (300 K), an information production of 39.3 Tb/s would require equivalent energies. The implications of this for telecom efficiencies are still being worked out.

## 4. Discussion

Purpose is necessarily irreversible, and there exists today a growing interest in irreversible systems. For recent examples, Jeynes [37] reviews a standard approach to certain non-adiabatic systems, and Aslani et al. [38] discuss micro-rotation in (non-Newtonian) micropolar fluids (where a “Newtonian fluid” has an idealised viscosity and is therefore intrinsically irreversible).

Living things are characterised by purpose—at a minimum they want to survive. Although as humans we are very good at discerning purposes, it is generally assumed that talk of “purpose” is not properly “scientific”, for the very good reason that what we mean by “purpose” is inescapably metaphysical. And metaphysics (the metanarrative of physics) is necessarily inexpressible in physical terms.

However, we have found a way of mathematically expressing a cut-down version of “purpose” (that is, shorn of its metaphysical aspects: “*entropic purpose*”) that will certainly help to physically distinguish the animate from the inanimate, which we expect to shed light on critical issues of practical engineering concern related to assessments of ‘aliveness’, personality aspects of artificial intelligence (**AI**) systems, and tests to distinguish actual human actors from bots in a cybersecurity context.

Our *definition* of “*entropic purpose*” relies heavily on Shannon’s information metric, an important aspect of thermodynamics. That is, our treatment of *entropic purpose* derives from recent progress in the study of Quantitative Geometrical Thermodynamics (QGT) that represents a new and powerful approach to the understanding of system entropy, as expressed by a fully canonical Lagrangian-Hamiltonian formulation that treats information and entropy as mutually orthogonal (whose spacetime base axes are Hodge duals) in a holomorphic entity: *info-entropy*. QGT is a general formalism with wide-ranging consequences, in particular, that *entropy production* (the rate of entropy increase) is demonstrably an isomorph of *energy*, and both are Noether-conserved. Since information production (characteristic of life) is very closely related to entropy production, we can derive important results (presented here) on the mathematical characteristics of *entropic purpose*. Its application in a range of current AI-based technologies remains to be developed.

One important aspect to highlight is that intrinsic to the Euler-Lagrange formalism of Equation (2) is the teleological character of the purposive Lagrangian. This is an important attribute that is essential to any discussion of *entropic purpose* since purpose is necessarily orientated to the future. As is already well recognised for the Principle of Least Action [1,2], there is a strong teleological element to its interpretation (the PLA was after all the original stimulus for the development of the mathematical apparatus of the kinematic Lagrangian and the associated Euler-Lagrange equation). For example, in adopting a trajectory across spacetime, a geometric light ray apparently ‘chooses’ in advance which trajectory (with its initial angle of departure) will ensure the least time is taken to reach the ‘intended’ destination. The purposive Lagrangian is equally teleological for the same reason. That is to say, the *entropic purpose* of a system is minimised (according to the variational calculus of the Euler-Lagrange equation) by its trajectory across the complex temporal plane, in accordance with the amount of information created by the process.

Thus, the *entropic purpose* of a system is equivalent (in metric) to the quantity of information created by the system. On the one hand, the creation of such information is straightforwardly and simply measured using the Shannon metric; which might imply that the measurement of entropic purpose as a distinct quantity is therefore superfluous or even a tautology. However, this is to overlook the essential feature of purpose which is its future orientation. The Shannon metric does not consider the causality, future orientation, or even the teleological nature of the information it is measuring: Shannon assumed that the information already existed. However, when considering the (ex-nihilo) creation of such information, then its (entropic) purposive nature comes to the fore and needs to be explicitly considered. In this paper, we show that the creation of such Shannon information can be described using a purposive Lagrangian that obeys the Euler-Lagrange equation so that its teleological or future-orientation characteristics are intrinsic; albeit implicit and also frequently ignored in epistemological considerations of the variational calculus. Here, we have only proven that such a theoretical Lagrangian framework for *entropic purpose* exists in a coherent mathematical formalism and that it successfully describes the creation of Shannon information. The explication of an appropriate (or specific) purposive Lagrangian for any particular information-creating system is the subject of future research. Here, we are only indicating the fact that such a purposive Lagrangian can be coherently defined, see Equation (8); and that it is also consistent with and fits into the current physical and mathematical state of knowledge.

Another critical insight is that the complex (energy) Hamiltonian of a system ([3]) is equivalent to the complex conjugate of the system’s entropy production. That is, the real entropy production is isomorphic to the imaginary energy, and the imaginary entropy production is isomorphic to the real energy; such a relation is predicated on a description of time in the complex temporal plane, thereby providing a consistent physical description of thermodynamically reversible and irreversible processes. This means that as a system evolves, it describes a temporal trajectory across the complex temporal plane that satisfies this description of *entropic purpose*.

QGT was originally developed to treat Maximum Entropy (**MaxEnt**) entities, which are necessarily stable in time, ranging from the sub-atomic to the cosmic, from nuclear isotopes and DNA to black holes. Curiously, being “stable” does not necessarily mean being “unchanging”, since black holes are MaxEnt (actually, they are the archetypal MaxEnt entity) but they also necessarily grow. Surprisingly, in the QGT formalism, it is the *geometry* of MaxEnt entities that incorporates the Second Law.

Now, whenever living entities are not dormant (that is, when they are actively exhibiting purposes) they cannot be MaxEnt—rather, they are in a far-from-equilibrium state, as discussed authoritatively by Pressé et al. [33] and Pachter et al. [39], and note that Parker and Jeynes [22] have shown that what these authors call “caliber” is identified with “exertion” in QGT, where “exertion” is defined as a line integral on an appropriate (entropic) Lagrangian. Here, we have found a temporal (rather than a geometrical) entropic Lagrangian to express this case (naturally limited to *entropic* purpose). The (non-trivial) issue here is that in the context of complex time (enabling a unified approach to reversible and irreversible processes), a valid Lagrangian is fully complexified: its real and imaginary components essentially represent respectively the energetic and entropic aspects of any such system.

It is also relevant to note that the time *T* associated with the physical process of producing (creating) information is also larger than if the process created zero information, where the conventional elapsed time (for the non-creation of information) would be the thermodynamically reversible time *t*. That is to say, arguably, the presence of an information-producing phenomenon causes time to dilate (lengthen). The implications of this in the physical world are intriguing. A black hole (or, indeed, a supermassive BH) which is arguably the most entropic as well as the most entropy-producing (and thereby, energetic) object in a galaxy should therefore cause time dilation as per the discussion centred on Figure 2. Of course, this is already well known from General Relativity on account of the BH’s mass. However, whether there is an additional effect on the dilation of time due to a BH’s entropy production (over and above that due to its mass), or whether the time dilation due to entropy production is simply an alternative (thermodynamic description) but still the same (i.e., an exactly equivalent), explanation for time dilation due to the presence of mass is the subject of further research.

Similarly, as conscious beings producing both entropy and information, we are all familiar with the passage of time. But could the passage of time be variable due to differing amounts of entropy production being associated with different living entities? Again, this is a fundamental question that requires additional research.

Scientists have been claiming for well over a century that purpose is essentially illusory, which seems to be in gross contradiction to our common sense; and one would not expect such a long and well-founded tradition to be easily overturned. However, the quantitative treatment of entropy is now at last promising useful progress, since we have shown that an *entropic purpose* can be expressed in properly physical terms: we expect that this will have far-reaching implications for the study of AI and information systems.

## 5. Summary and Conclusions

Inanimate things do not have purposes. But animate things (which do have purposes) are both real and material so physics should apply to them. Therefore, physics must incorporate quantities (defined impersonally of course) that in some sense look like “purposes”, and that can in principle be used to impersonally distinguish animate from inanimate things.

We have defined the “*entropic purpose*” of a heavily idealised system using the formalism of QGT (Quantitative Geometrical Thermodynamics), showing that systems with zero information production also have zero entropic purpose.

This has been possible only because QGT can be expressed in a fully general way which depends on the full complexification of the formalism [3], allowing us to bring to bear the powerful mathematics of complex analysis. It is noteworthy that Ivo Dinov’s group in Michigan also uses complex time (“kime”) in a 5-D “spacekime” to solve big data problems (see for example Wang et al. [31]). This fully general (complexified) formalism also treats reversible and irreversible systems commensurately—and living systems are all irreversible! This is the answer to the longstanding Loschmidt Paradox that has puzzled physicists since Ludwig Boltzmann replied to Joseph Loschmidt (see Olivier Darrigol’s helpfully commented translation [40]): how does the real (irreversible) world ‘emerge’ from the (apparently all reversible) equations of physics? Our QGT resolution shows constructively that physics coherently treats reality, whether or not it is reversible.

It is surprising how much useful physics can be done using only reversible theories (perhaps with some perturbation theory), but this QGT treatment underlines that it is the reversible theories that are all approximations: fundamentally, they are idealisations from the irreversible generality! This is now starting to be recognised with an increasing interest in non-Hermitian systems (recently reviewed [37]); and of course, Ilya Prigogine’s “Brussels-Austin Group” has long been systematically approaching non-equilibrium thermodynamics.

QGT is an analytical theory. So far, we have treated only simple (high symmetry) systems that yield readily to an analytic approach, having previously shown very simple (and demonstrably correct) QGT treatments of known systems that are nearly intractable using traditional approaches (including DNA and spiral galaxies [13]; fullerenes [28]; alpha particle size [29]; and free neutron lifetime [30]).

Here, we have demonstrated that mainstream physics also applies in a non-trivial way even to living beings: in particular that it is now possible in principle to recognise (at least in part) the purposefulness of life.

Specifically, we have proved that a “purposive Lagrangian” exists and, moreover, that the “*entropic purpose*” of a system may be measured by the Shannon information it creates. That is, it is not necessary to construct the purposive Lagrangian to quantify the *entropic purpose*.

## Data Availability

The original contributions presented in this study are included in the article material. Further inquiries can be directed to the corresponding author.

## Appendix A. Principle of Least (Entropic) Purpose

We confirm, using the same method as in Appendix C of PJ19 [13], that the Euler-Lagrange equation (Equation (9) is indeed satisfied for the trajectory *l* (associated with the sample purposive Lagrangian *L_P_* of Equation (8) across the complex plane (see Figure 2). We note that the path of the trajectory is particularly entailed by the time *T* required to produce a given amount of information, such that the calculus of variations analysis must use *T* as the key differentiating variable.

Note that this variational principle (which mirrors the *Principle of Least Action*) concerns the scientific quantity of “*entropic purpose*”, not the philosophical idea of “*purpose*”.

The two orthogonal dimensions in the complex temporal plane (see Figure 2) are the (reversible time) *t* axis and the (irreversible time) *τ* axis. We consider the expression for a generalised holomorphic trajectory across the temporal *z* = −*τ* + i*t* plane (such a trajectory is analogous to a double-helix in QGT), where in our formalism we also continue to explicitly conform to the handedness of the *z*-plane:

(A1a) $$l\equiv -Ze^{i\kappa t}\hat{\underline{\tau }}+iZe^{i\kappa \tau }\hat{\underline{t}}$$

(A1b)
$$\;l\equiv -Z_{0}e^{-\Lambda T}e^{i\kappa T\cos\theta }\hat{\underline{\tau }}+iZ_{0}e^{-\Lambda T}e^{i\kappa T\sin\theta }\hat{\underline{t}}\;\;\;\;\;$$

(A2a)
where t≡Tcos⁡θ and τ≡Tsin⁡θ

(A2b)
$$and\;Z\equiv Z_{0}e^{-\Lambda T}$$

Equation (A1a) *approximately* represents a double-helix with an axis located along the temporal *T* direction which is rotated at an angle θ in the complex temporal plane (see Figure 2). When θ = 45° then the double-helical trajectory description is equal to the ‘simple’ equation, Equation (8) of PJ19 [13] (where using QGT concepts, the τ axis is equivalent to the *x*_1_ axis, and *t* is equivalent to the *x*_2_ axis, while *T* is equivalent to *x*_3_).

When θ = 0 or 90° then we only have a single-helix (rather than a double-helix) trajectory geometry with the additional value of *Z* along one of the temporal axes. The values of *Z* for these two extreme cases θ = 0, 90° both act as (constant) offsets which, when invoking the (differential) canonical relations (the Euler-Lagrange, Lagrangian/Hamiltonian, or the Cauchy-Riemann equations), differentiate to zero.

In Equation (A2b) (for an information-producing system), we define the system’s characteristic holographic temporal parameter *Z* (equivalent to the spatial holographic radius in QGT) to vary with the parameter Λ along its temporal *T* axis. This is equivalent to the diminution of radius in a double logarithmic spiral along the axis of the spiral (see Appendix B of PJ19, Equation (B.32) *passim* [13]). The parameter *Z*_0_ represents the characteristic (holographic) time at the beginning of the (possibly information-producing) process.

The holomorphic trajectory *l* has a description in the (Euclidean) temporal plane, with the two co-ordinate functionals *z*_t_, *z*_τ_ describing the evolution of the holomorphic trajectory across the complex time plane:

(A3a) $$l\equiv -z_{\tau }\hat{\underline{\tau }}+iz_{t}\hat{\underline{t}}\;$$

(A3b)
$$z_{t}=Ze^{i\kappa T\sin\theta }$$

(A3c)
$$z_{\tau }=Ze^{i\kappa T\cos\theta }$$

### Appendix A.1. Conjugate Parameters in Hyperbolic Space

The entropic Lagrangian is defined in hyperbolic (not Euclidean) space (see Equation (7c) earlier, and Equation (9) *passim* of PJ19 [13]), and the hyperbolic position *q* is given by the transformation:

(A4) $$q_{n}\equiv Z\ln\frac{n}{Z}$$

where *n* ∈ {*t*,*τ*} and *Z* (Equation (A2b)) is the instantaneous temporal holographic parameter. Taking the two co-ordinate functionals *z*_t_, *z*_τ_ in turn using the Equation (A3a)–(A3c), and substituting them for *n* in Equation (A4), we transform into the following two hyperbolic temporal co-ordinates:

(A5a) $$q_{t}\equiv Z\ln\frac{t}{Z}=iZ\kappa T\sin\theta$$

(A5b)
$$q_{\tau }\equiv Z\ln\frac{\tau }{Z}=iZ\kappa T\cos\theta$$

We then differentiate the expressions for *q_n_* (Equations (A5a,b) by the axial temporal parameter *T*, to obtain the (conjugate) hyperbolic velocities $q_{t}^{\prime }$ and $q_{\tau }^{\prime }$ (which are proportional to the momenta):

(A6a) $$\frac{\partial q_{t}}{\partial T}\equiv q_{t}^{\prime }=i\kappa Z_{0}e^{-\Lambda T}\sin\theta \left(1-\Lambda T\right)=i\kappa Z\sin\theta \left(1-\Lambda T\right)$$

(A6b)
$$\frac{\partial q_{\tau }}{\partial T}\equiv q_{\tau }^{\prime }=i\kappa Z_{0}e^{-\Lambda T}\cos\theta \left(1-\Lambda T\right)=i\kappa Z\cos\theta \left(1-\Lambda T\right)$$

The entropic Euler-Lagrange equation that describes the *Principle of Least (entropic) Purpose* is then given by:

(A7) $$\frac{d}{dT}\frac{\partial L_{P}}{\partial q_{n}^{\prime }}-\frac{\partial L_{P}}{\partial q_{n}}=0$$

### Appendix A.2. Obtaining the Purposive Lagrangian

We call the Lagrangian appropriate to the complex temporal plane the “Purposive Langrangian” *L_P_*.

Lagrangians (including *L_P_*) are obtained quite generally in hyperbolic (information) space from PJ19 (Equations (C23)–(C25) of Appendix C [13]), comprising a ‘kinetic’ and a ‘potential’ term:

(A8) $$L_{P}=\sum_{n=t,\tau }{cm}_{S}\ln q_{n}^{\prime }-V_{P}\left(q_{n}\right)$$

A general derivation of the purposive potential terms (corresponding to an inverse-square law force relationship in Euclidean space) is given in PJ19 Appendix B (summarised in Equation (C24) [13]):

(A9a) $$V_{P}\left(q_{t}\right)=-{cm}_{S}\left[\Lambda T-\ln\left(1-\Lambda T\right)\right]-m_{S}\ln K_{t}$$

(A9b)
$$V_{P}\left(q_{\tau }\right)=-{cm}_{S}\left[\Lambda T-\ln\left(1-\Lambda T\right)\right]-m_{S}\ln K_{\tau }$$

The purposive Lagrangian is therefore given by:

(A10a) $$L_{P}={cm}_{S}\left(\ln q_{t}^{\prime }+\ln q_{\tau }^{\prime }\right)+2{cm}_{S}\left[\Lambda T-\ln\left(1-\Lambda T\right)\right]+{cm}_{S}\ln K_{t}K_{\tau }$$

where $K_{t}{\;and\;K}_{\tau }$ are constants of integration and where *m_S_* is the entropic mass as before (see Equation (7b), *passim*). Substituting Equation (A6a,b) into Equation (A10a), it is straightforward to rearrange and additionally derive:

(A10b) $$L_{P}=cm_{S}\ln\left(\sin2\theta \right)+cm_{S}\ln\left(-\kappa^{2}Z_{0}^{2}/2\right)+cm_{S}\ln K_{t}K_{\tau }$$

as per Equation (12) in the main text.

### Appendix A.3. Confirming the Variational Properties on the Reversible t Axis

Partial differentiating the purposive Lagrangian *L_P_* of Equation (A10a) with respect to the differential (hyperbolic velocity) quantity $q_{t}^{\prime }$:

(A11a) $$\frac{\partial L_{P}}{\partial q_{t}^{\prime }}=\frac{{cm}_{S}}{q_{t}^{\prime }}$$

Substituting Equation (A6a) into Equation (A11a) we have:

(A11b) $$\frac{\partial L_{P}}{\partial q_{t}^{\prime }}=\frac{{cm}_{S}e^{\Lambda T}}{i\kappa Z_{0}\sin\theta \left(1-\Lambda T\right)}$$

Differentiating with respect to *T* we have:

(A11c) $$\frac{d}{dT}\frac{\partial L_{P}}{\partial q_{t}^{\prime }}=\frac{{cm}_{S}\Lambda e^{\Lambda T}\left(2-\Lambda T\right)}{i\kappa Z_{0}\sin\theta {\left(1-\Lambda T\right)}^{2}}$$

The second term of the Euler-Lagrange equation, Equation (A7) is found as follows, using Equations (A8) and (A9a) as the preceding parts of Equation (A10):

(A11d) $$\frac{\partial L_{P}}{\partial q_{t}}=-\frac{\partial V_{P}}{\partial q_{t}}=-\frac{\partial V_{P}}{\partial T}\frac{\partial T}{\partial q_{t}}=cm_{S}\left(\Lambda +\frac{\Lambda }{1-\Lambda T}\right)\frac{1}{q_{t}^{\prime }}=\frac{{cm}_{S}\Lambda e^{\Lambda T}\left(2-\Lambda T\right)}{i\kappa Z_{0}\sin\theta {\left(1-\Lambda T\right)}^{2}}$$

Thus, by inspection (Equations (A11c,d)), the Euler-Lagrange equation for the *n* = *t* time co-ordinate is satisfied:

(A11e) $$\frac{d}{dT}\frac{\partial L_{P}}{\partial q_{t}^{\prime }}-\frac{\partial L_{P}}{\partial q_{t}}=0$$

### Appendix A.4. Confirming the Variational Properties on the Irreversible τ Axis

Similarly, for the other *n* = *τ* temporal coordinate. Partial differentiating the purposive Lagrangian *L_P_* with respect to $q_{\tau }^{\prime }$:

(A12a) $$\frac{\partial L_{P}}{\partial q_{\tau }^{\prime }}=\frac{{cm}_{S}}{q_{\tau }^{\prime }}$$

Substituting Equation (A6b) into Equation (A12a) we have:

(A12b) $$\frac{\partial L_{P}}{\partial q_{\tau }^{\prime }}=\frac{cm_{S}e^{\Lambda T}}{i\kappa Z_{0}\cos\theta \left(1-\Lambda T\right)}$$

Differentiating with respect to *T* we have:

(A12c) $$\frac{d}{dT}\frac{\partial L_{P}}{\partial q_{\tau }^{\prime }}=\frac{{cm}_{S}\Lambda e^{\Lambda T}\left(2-\Lambda T\right)}{i\kappa Z_{0}\cos\theta {\left(1-\Lambda T\right)}^{2}}$$

The second term of the Euler-Lagrange equation, Equation (A7) is found as follows, using Equations (A8) and (A9b):

(A12d) $$\frac{\partial L_{P}}{\partial q_{\tau }}=-\frac{\partial V_{P}}{\partial q_{\tau }}=-\frac{\partial V_{P}}{\partial T}\frac{\partial T}{\partial q_{\tau }}={cm}_{S}\left(\Lambda +\frac{\Lambda }{1-\Lambda T}\right)\frac{1}{q_{\tau }^{\prime }}=\frac{{cm}_{S}\Lambda e^{\Lambda T}\left(2-\Lambda T\right)}{i\kappa Z_{0}\cos\theta {\left(1-\Lambda T\right)}^{2}}$$

Thus, by inspection (Equations (A12c,d)), the Euler-Lagrange equation for the *n* = *τ* time co-ordinate is satisfied:

(A12e) $$\frac{d}{dT}\frac{\partial L_{P}}{\partial q_{\tau }^{\prime }}-\frac{\partial L_{P}}{\partial q_{\tau }}=0$$

confirming that the trajectory *l* (Equations (A1)) obeys the variational principle (Equation (A7)), as required.

## Appendix B. Analyticity of the Purposive Lagrangian in the Complex Temporal Plane

We briefly indicate the validity of the Cauchy-Riemann equations as applied to the complexified purposive Lagrangian *L_P_* in the complex temporal plane, $z\equiv i\left(t+i\tau \right)$. In particular, we assume that the purposive Lagrangian can be expressed by a pair of purely real functions, *F* and *G*, which together form an analytic function, Σ:

(A13) Σ=F+iG

Note that it is a standard result of complex analysis that *F* and *G* are mutually Hilbert Transforms. For the *z* =−*τ* + i*t* plane, the relevant Cauchy-Riemann equations are then:

(A14a) $$\frac{\partial F}{\partial t}=-\frac{\partial G}{\partial \tau }$$

and

(A14b) $$\frac{\partial F}{\partial \tau }=\frac{\partial G}{\partial t}$$

Employing the purposive Lagrangian of Equation (12), substituting in Equation (13) and only considering the varying part of *L_P_*, we have Equation (14a):

(A15a) $$L_{P}=cm_{S}\ln\left(\sin2\theta \right)$$

This function is purely real and therefore cannot constitute an analytic function. To make it analytic, we must complexify it, therefore, requiring the Hilbert Transform of sin2*θ* (i.e., −cos 2*θ*). However, the appropriate complexified version of the purposive Lagrangian appropriate to the handedness of the *z*=−*τ* + i*t* plane is then given (with some simple algebraic manipulation) by:

(A15b) $$L_{P}=cm_{S}\ln\left(\sin2\theta +i\cos2\theta \right)$$

(A15c)
$$L_{P}=cm_{S}\ln\left(ie^{-2i\theta }\right)=cm_{S}\left(-2i\theta +i\frac{\pi }{2}\right)$$

We ignore the constant (d.c. offset) aspect to the purposive Lagrangian since it differentiates away when the Cauchy-Riemann conditions are applied; then using Equation (13), we have (using the identity $\tan^{-1}x\equiv \frac{i}{2}\ln\frac{i+x}{i-x}$):

(A15d) $$L_{P}=-2icm_{S}\theta =-2icm_{S}\tan^{-1}\left(\tau /t\right)$$

(A16)
$$L_{P}=cm_{S}\ln\left(\frac{t-i\tau }{t+i\tau }\right)$$

We exponentiate Equation (A16) to make for easier manipulation; noting that the *exp* function is represented by a ‘well-behaved’ power series (no poles) that is unconditionally stable; meaning that the analytic properties of *L_P_* are not changed by the exponentiation. In this case the analytic function of interest is now given by:

(A17) $$\Sigma \equiv e^{\frac{L_{P}}{cm_{S}}}=\frac{t-i\tau }{t+i\tau }=\frac{t^{2}-\tau^{2}-2it\tau }{t^{2}+\tau^{2}}$$

hence identifying the real and imaginary functions in Equation (A13):

(A18a) $$F\equiv \frac{t^{2}-\tau^{2}}{t^{2}+\tau^{2}}=\frac{t^{2}-\tau^{2}}{T^{2}}$$

(A18b) $$G\equiv -\frac{2t\tau }{T^{2}}$$

where we note that the quantity $\sqrt{t^{2}+\tau^{2}}$ corresponds to the system invariant *T*, this being the empirically measured *least time* for the information production. The temporal derivatives of the Equations (B18a,b) are then simply given by:

(A19a) $$\frac{\partial F}{\partial t}=\frac{2t}{T^{2}}$$

(A19b)
$$\frac{\partial G}{\partial \tau }=-\frac{2t}{T^{2}}$$

(A19c)
$$\frac{\partial F}{\partial \tau }=-\frac{2\tau }{T^{2}}$$

(A19d)
$$\frac{\partial G}{\partial t}=-\frac{2\tau }{T^{2}}$$

Thus the Cauchy-Riemann relations of Equations (A14a,b) are satisfied by Equations (A19a–d), demonstrating the analyticity of the (complexified) purposive Lagrangian *L_P_* in the complex temporal *z*-plane.

## Appendix C. The Legitimacy of Teleology

### Appendix C.1. Life Is Purposive

It is generally assumed that a concept such as ‘*purpose*’ is inevitably anthropomorphic (and therefore ‘unscientific’). However, the more limited idea of ‘*entropic purpose*’ may be defined impersonally in much the same way that Claude Shannon [11] famously defined his eponymous ‘information’ impersonally. Noting Sara Walker’s assertion in her 2017 review that a “*key challenge is identifying the properties of living matter that might distinguish living and non-living physical systems*” [41], we point out that all living entities must have a non-zero *entropic purpose*.

Of course, this takes for granted the old observation that “*life is a manifestation of the Second Law of Thermodynamics*” [42]. That life is a “low entropy” state has been known at least since Schrödinger’s seminal 1944 book, “*What is Life?*” [43], in which he also insisted that a characteristic of life is that it creates “*order from disorder*”. This characteristic is necessary but not sufficient since subsequently many inanimate systems have been found where an entropy flow brings “order from disorder” (one of the simplest being the Bénard cell, analysed by Schneider and Kay [42]).

But it is also well known that living organisms are “purposeful”, distinguishing them from inanimate systems. Lynn Margulis and Dorion Sagan say (and elaborate at length) [44]: “*When offered a variety of foodstuffs … mobile microbes make selections—they choose*”. They also emphasise Samuel Butler’s contribution (Butler was Darwin’s contemporary): “*Retreating from Darwin’s neo-Newtonian presentation of organic beings as “things” acted on by “forces,” Butler presented sentient life as making numberless tiny decisions … the sum effect of little purposes*” where it is the “little purposes” of the small organisms that cumulatively change the face of the planet. They say, “*In Butler’s view all life … is teleological; that is, it strives. Butler claimed that Darwinians missed the teleology, the goal-directedness of life acting for itself. In throwing out the bathwater of divine purpose, Darwin discarded the baby of living purposefulness*”. They conclude, “*We agree with Butler that life is matter that chooses*”, also agreeing with Niels Bohr: “… *Bohr … contended that … there was a need for description that includes “purposiveness*”. Mark Bedau [45] proposes and defends the idea of “supple adaptation” as the defining characteristic of life, saying: “*The notion of propriety [appropriateness] involved in supple adaptation is to be understood teleologically. A response is ‘appropriate’ only if it promotes and furthers the adapting entity’s intrinsic goals and purposes …*” Bedau seems to be using his term “supple adaptation” as a near-synonym of (or periphrasis for?) “purpose”. We will drop these circumlocutions.

Corning et al. point out [46] that James Shapiro has shown “burgeoning evidence that the genome is in fact a ‘two-way read-write system’” quoting Shapiro as saying, “The capacity of living organisms to alter their own heredity is undeniable”. This has long been known, if rarely acknowledged: McFadden and al-Khalili [47] already showed in 1999 how to interpret observations on E-coli made in the 1980s as purposive (they called it “adaptive mutation”).

In §3, we show how to define “*entropic purpose*” as a legitimate concept of physics. It is necessary to add that just as the 2nd Law is fundamental, so we have also shown that thermodynamics is *not* “emergent” from statistical mechanics (contrary to much scientific opinion today). Not only do we show in a Quantitative Geometrical Thermodynamics (**QGT**) treatment that the entropic Partition Function can be obtained from the entropic Liouville Theorem (Equation (16) of Parker and Jeynes 2021 [27]), but we also show that QGT applies directly to *small* systems (for example the alpha particle, with only three degrees of freedom: Parker et al. 2022 [29]). This contradicts Orly Shenker’s assertions, not only that there is a “*statistical mechanical underpinning of the notions of probability and entropy*”, but also that “*information plays no fundamental role in these*” [48]. Note that *probability* itself is a physical (rather than only a logical) concept [49].

Léon Brillouin’s intuition long ago that information and entropy were two sides of the same coin remains sound [50]. An impersonal “entropic purpose” can be defined just as can an impersonal “Shannon information”.

### Appendix C.2. Aristotelian Teleology

“*Purpose*” is famously a central element of Aristotelian physics: Mariska Leunissen emphasises the teleological implications of the *form* of entities, saying that “the *way* form is a ‘principle’ (αρξη [*archē*]) is by being an ‘end’ (τελοσ [*telos*])” [51] (emphasis original). Of course, contrary to Aristotle’s view of what physics is about, we are correct today in regarding the “why” questions as not being proper to physics (so that Sara Walker’s *Phil. Trans.* summary [52] does not mention “purpose”, and neither does her *Rep. Prog. Phys*. review [41]): nevertheless, it seems that we are now finding that Aristotle is not as wrong as we thought he was. Carlo Rovelli [53] calls Aristotle’s physics “*sound*” (referring to its internal coherence), saying that his was: “*the first systematic physics we know of, and it’s not bad at all*” (*ibid*. p.27f). George Ellis’ review helpfully explains the relevance today of Aristotle’s “Four Causes” [54]. We will return to this.

We have already shown that the *form* of an entity does indeed tell us much, with QGT correctly determining (without any recourse to quantum mechanics) both the size of the alpha particle [29] and also the half-life of the free neutron [30]: indeed the variational *Principle of Least Action* (which both Max Planck and David Hilbert understood as underpinning all of physics: see [1,2]) has always been thought to have teleological aspects (which the practice of modern science has done its best to eliminate, or at least minimise: see the review by Michael Stöltzner [2]). James Allen shows that Aristotle considers that “*chance events*” themselves constitute “*proofs of the existence of natural teleology*” [55] (on well-argued grounds similar to “*the exception proves the rule*”), and we have shown the commensurability of the causal and the acausal (PJ23 [3]).

Charlotte Witt [56] explores Aristotle’s teleology in Book II of the *Physics*, arguing that artefacts do indeed have “intrinsic ends” and “proper functions” like natural beings (just as Aristotle says they do, and therefore that they are ontologically comparable), and consequently that Aristotle’s analogy between art and nature is neither mistaken nor misleading in principle: we have shown that physics cannot be *understood* without (usually tacitly) discerning its poetry [9].

Moreover, we have also shown that the *form* of spiral galaxies can be regarded (apparently teleologically) as generated by a variational principle (Parker and Jeynes 2019 [13]) such that they have both local and non-local characteristics. Note that spiral galaxies are too large for any feedback mechanism to account for their form, such an account must be non-local (the Milky Way is 100,000 light-years across, for example). Margaret Scharle underlines Witt’s conclusion about the preeminent importance of *form*, saying: ‘*Aristotle brings together the arguments of* Physics ii.1 *(that form is more nature than matter) with the first argument of* Physics ii.8 *(that nature is ‘for-the-sake-of’ something) to conclude that “form must be the cause in the sense of that-for-the-sake-of-which* [η ου ενεκα, *ē ou eneka*]*”* ’ [57]. We would not argue like this today, but Aristotle’s insight into *form* itself intrinsically being a *cause* (in some sense) seems to be consistent with much modern work (including ours); in particular with regard to the non-local natures of such relations.

It is commonly thought today (*contra* Aristotle) that Nature is purposeless: so George Ellis asserts [58] that “*purposeless physics underlies purposeful life*” (commenting on Sharma et al.’s “assembly theory” [59]). It is also commonly thought that everything that is, is material; but Aristotle (in his *Physics*: see [51]) explicitly argues (against his materialist predecessors Democritus and Empedocles) that nature is *purposeful*, although his terms diverge strongly from ours. The title of his book, “Φυσικὴ ἀκρόασις”[*physikē akroasis*] (literally “listening to nature”, probably meaning “lectures on nature”), and his argument is subtle and multifaceted. No wonder he still has commentators!

Aristotle’s “Four Causes” (see Ellis 2023 [54]) were remembered in Europe because Thomas Aquinas was able in the 13th century to Christianise the pagan philosopher for the new universities (Paris in particular). The mediaeval scholastics soon surpassed Aristotle in physics [60], but the Thomist reading of Aristotle was made canonical by the (16th century) Council of Trent. Galileo famously railed against the newly fashionable Aristotelian dogma [61]: it is an irony of the history of science that in the 17th century this dogma was buttressed by Enlightenment humanism which loved the (Greek and Roman) classics and detested the dry logic of the scholastics: the insult “dunce” (a corruption of “Duns Scotus”, the great scholastic logician) dates from the 16th-century Dissolution of the Monasteries in Britain, when the major libraries were also pillaged, including throwing many scholastic manuscripts to the wind. But it is now well known that Galileo was indebted to (among others) the renowned 14th-century scholastic Jean Buridan [62,63].

However, Aristotle’s “cause” (a Latin word from Aquinas and his predecessors, “αιτια” [*aitia*] in Aristotle’s classical Greek—hence *aetiological*) is rendered in English better as “*explanation*” rather than what we now think of as “*cause*”. This also throws into sharp relief the deeply human way Aristotle goes about his enquiry (contrasting strongly with the impersonal method we now think proper for science) which can be seen by the way he opens the *Physics* (here we follow James Lennox’s account [64] and also see the extensive discussion in Jeynes and Parker 2024 [65]) with a discussion of the method [μεθοδος, *methodos*] proper to obtaining scientific knowledge [επιστημη, *epistēmē*] in a scientific enquiry [ϕυσιν ιστοριας, *physin istorias*]. The word “μεθοδος” is formed from the noun οδος (a *road*) and the prefix μετα (in this context having the force of “*in quest of*”), thus meaning “*a path taken in pursuit of …”* (in this case, knowledge). Plato already used μεθοδος in the *Republic* speaking of “*the dialectical method*” [η διαλεκτικη μεθοδος, *ē dialektikē methodos*] as the only way to advance to first principles. Consequently (and note that if the “first principles” are the *beginning* of physics, then the “dialectical path” to them must be a *metaphysical* one), Aristotle understood very well that the scientific method is necessarily metaphysical, a conclusion we have reached independently since the *meaning* of the terms used in the discussion cannot be established by the discussion itself [9].

Annie Crawford argues that “*teleological language cannot be ‘merely’ metaphor*” [66] because were it not in fact essential, then the biologists (who hate teleological language: see §1.6) would have replaced it: she says, “*That the language of purpose and design persists to annoy so many committed naturalists is itself evidence that the language of teleology is important to the study of life*”. Philip Ball [67] (p. 363) quotes her: “*what makes a creature* **alive** *is its teleological process: a material form animated by the striving of a unique being to become and remain itself*” (emphasis in original). Ball comments (*ibid*.) “*Pretending agency doesn’t exist is asking for trouble*”.

The reality of metaphors is well-known: Iris Murdoch commented long ago [68], “*Metaphors are not merely peripheral decoration or even useful models, they are fundamental forms of the awareness of our condition … it seems to me impossible to discuss certain kinds of concepts without the resort to metaphor, since the concepts are themselves deeply metaphorical and cannot be analysed into non-metaphorical components without a loss of substance*”. Crawford makes essentially the same point, not citing Murdoch but citing Owen Barfield’s early work (Poetic Diction, 1928), as do also Jeynes et al. [9]. Potter and Mitchell [69] discuss “*agent causality in an entirely naturalistic and non-mysterious way*”. And of course, only agents have purposes. It seems that this approach to realism (that Karen Barad [70] calls “*agential realism*”) is becoming quite widely held.

### Appendix C.3. Background to Entropic Purpose

George Ellis regards *purpose* as an “emergent” property of nature [71]. An example of this emergence might be marine predators which have been shown in foraging to exploit certain types of ‘*Lagrangian coherent structures*’ (that is, impersonal physics: see for example the 2022 review by Sergey Prants [72]). Nevertheless, this is demonstrably a learnt—not an automatic—behaviour (although how individuals learn to identify these mathematical structures remains a mystery): Grecian et al. 2018 [73] have shown a substantial difference between mature birds and juveniles in a study of gannets using ‘*hidden Markov models*’ (more impersonal physics!) to characterise three labelled sub-behaviours (travelling, foraging, resting; and see Connors et al. 2021 [74] for similar work on albatrosses). The interplay between non-local characterisations of system behaviour and the (very local) series of actions taken by these birds is indicative of the proper way to regard the relationship, on the one hand between the (non-local) complex behaviour of systems in which entropy is flowing, and on the other hand the specific (local) actions of living entities in those systems. It is these local actions (the birds *choosing* which way to go) that we regard as introducing (creating) Shannon information (and entropic purpose) into the system.

Thus, we show that *entropic purpose* is fundamental to the description of the phenomena: the idea of ‘emergence’ is redundant except in the weaker form established by Denis Noble [75] of ‘downward causation’ in the sense of non-local system constraints (using his seminal modelling of the heartbeat). Non-local system constraints also include the variational Principles—*Least Action*, *Maximum Entropy*, *Least (entropic) Purpose* (for which see Appendix A here), etc.—and are therefore natural to the present treatment.

Ilya Prigogine and his school have already established the fundamentally important yet very surprising result that the flow of entropy (“entropy production”) is the precondition for the establishment of ordered states in (otherwise) chaotic systems [76]. This is why we assert that one necessary (but not sufficient) condition for an entity to be considered living is that its entropy production must be non-zero. We already have substantial results for the quantity *entropy production*, having shown that it is Noether-conserved [20], and having confirmed our previous calculation for black hole entropy production [3] (noting that in our QGT formalism [13], black holes are also the simplest MaxEnt entities with non-zero entropy production).

### Appendix C.4. Information, Entropy and Noise

We have shown (using our QGT formalism) that *entropy* and (Shannon) *information* are very closely related—their basis vectors are actually Hodge duals [13]—such that a holomorphic quantity “info-entropy” may be defined which has the interesting mathematical properties we have cited. We have shown further [3] that *action* and *entropy* are equally closely related (yielding the holomorphic quantity “actio-entropy”) although, taking advantage of the properties of analytic continuation, this is only defined in a fully complexified treatment in which time is also complexified. This treatment shows that the (complex) entropy production is simply the (Wick rotated) complex conjugate of the (complex) energy Hamiltonian (in holographic natural units: see Equation (23c) of PJ23 [3]). The real and imaginary components of these complex quantities are also related through Hilbert transforms and the whole discussion (using complex analysis) is set up as the interplay between the local and the non-local, as well as between causality and acausality, reversibility and irreversibility, and between what is considered “real” and what is “imaginary” in complex spacetime, where we are free to choose the spacetime metric most convenient for the application (as Roger Penrose also insists in *Road to Reality* [15] §13.8).

Shannon’s theory of communication makes a *metaphysical* distinction between information and noise (which are physically indistinguishable); information is assumed to arise from human needs whereas noise arises randomly (acausally). The key distinction between a string of noisy bits and a string of information bits is whether algorithms exist, both at the origin (transmit end) and at the receive end of a communications channel, to code and subsequently decode the measured string such that noise may be robustly stripped from it to reveal only the original information. Of course, the purposive (intentional) information from such a communications channel is inseparable from our (human) purposes, but we are not here interested in this ‘personal’ aspect. Just as Shannon defined an impersonal metric to measure the “information content” of any signal, we impersonally define here a metric for the “entropic purpose” associated with the *generation* of said information.

The point here is that even though we cannot help but regard “*information*” anthropomorphically (*who* wants the information? *why*? what does it *mean*?), it may also be treated entirely impersonally as “*Shannon information*” [77], leading to Landauer’s conclusion “*Information is Physical*” [34,35,36]. Similarly, for “*purpose*”: Oliver Sacks writes movingly of human purpose (in the chapter, “*The World of the Simple*”) [78] with the example of Rebecca who said, “*I must have meaning*”. It is *we* who want (and need) purpose in our lives so this idea is irreducibly *personal*. Yet just as for *information*, we show that there is also a coherent and impersonal way of defining “*entropic purpose*” as a product of the principles of *causality* yet as applied (in agreement with Aristotle) in the context also of *acausality* (indeterminism).

John Toll (1956) [79] has given rigorous proof (in a complexified treatment) that the “dispersion relations” are logically equivalent to the existence of strict causality, and we (PJ23 [3]) have shown in detail that (Shannon) *information* is a form of causality. The (complex) dispersion relations very elegantly describe both local and non-local effects: as in the case of the (complex) optical refractive index for example, where absorption (dissipation at local sites) is represented by the imaginary component and refraction (a collective, or non-local, effect) by the real component. “Indeterminism” can be regarded as “the product of random processes” (or acausal phenomena), such as beta decay (which we have also treated thermodynamically [30]). Therefore, we expect that an impersonal “*entropic purpose*” can be readily defined (using QGT methods) as the generation (that is, the *cause*) of *Shannon information*. Clearly, the existence of *purpose* also implies the existence of *causality* relations. The agent whose purpose it is can be said to *cause* the (purposefully generated) results. Both purpose and the causality relations imply Time’s Arrow, that is, the Second Law of Thermodynamics (entropy increases as time passes).

This is germane to the machine learning and artificial intelligence (AI) communities, which are interested in *entropic causal inference*, its automation, and also its *causal discovery* (see for example Jaber et al. 2020 [80]) from ‘independent and identically distributed’ (i.i.d) data (see for example the 2016 review by Spirtes and Zhang [81]). We should note that the AI community is also very interested in “causal inference” (see Janzing et al. 2016 [82] for treatment in terms of Kolmogorov complexity); although in AI contexts, “cause” is generally used in the restricted sense of “Granger causality” (meaning that the “effects” can be *predicted* from the “cause”: see Quinn et al. 2015 [83] for the use of “directed information” [84] in these terms). Lombardi and López [85] have carefully distinguished “Integrated Information Theory”, **IIT** (which insists on the *meaning* of the information) from “Shannon information” (which explicitly does not). They assert that “*IIT is currently the leading theory of consciousness*” (although this is vigorously contested [86]) and state unequivocally that “*information is supported by a structure of causal links*”. We build on these insights in developing our proposal for the physical and impersonal representation of *entropic purpose*, while acknowledging that any wider view of *knowledge* is necessarily metaphysical [9].

We should point out that *actio-entropy* (PJ23, [3]) and *info-entropy* (PJ19, [13]) are closely related (since *action* and *entropy* are isomorphic), and we show that *entropic purpose* can therefore be represented impersonally as *introducing Shannon information*.

Regarding *entropic purpose* as a legitimately defined quantity amenable to scientific study is also relevant to the burgeoning field of drug discovery using natural language processing (NLP) methods to mine the existing (enormous) scientific literature for information to build reliable “knowledge graphs” of protein-gene relationships (that is, whether or not the protein binds to the gene or inhibits the gene action—see Jeynes et al. [87] for a recent example which underlines the extreme complexity of this effort). What is the “purpose” of the gene? of the protein? We (humans) are looking for methods to efficiently (that is, “cheaply”) find new drugs for particular purposes. In principle, including “*entropic purpose*” as a well-defined scientific entity must be helpful in underpinning such searches. We should add that the issue of assessing the accuracy of large databases is becoming more prominent: a recent example (Grime et al. [88]) is the demonstration that half of the metallo-proteins in the very widely used (and notionally authoritative) Protein Data Bank (www.wwpdb.org) may have misidentified metals.

The key aspect to point out is that all these important new developments in intelligence-based technology implicitly assume the (pre-)existence of Shannon information—a sequence of data symbols that has (metaphysical) meaning only to us—in contrast to a signal that is pure noise which, on its own, cannot be exploited in any way to extract useful meaning. Note, the use of a random number generator (RNG), such as those used in cryptography, is certainly an intelligent technology that is useful to secure communications—but its successful application ensures that eavesdroppers are unable to derive any meaning from intercepted data. In contrast, the authorised participants (at the transmit and receive ends) of the communications channel deploy their (intelligent) algorithms to strip off the ‘noise’ added by the RNG and perfectly reconstruct the originally transmitted information. The RNG creates no new information: it is simply used to allow only authorised actors to derive meaning from the transmitted Shannon information. And we define (impersonal) entropic purpose as the *creation* of such (impersonal) Shannon information.

### Appendix C.5. Emergence and Downward Causation

Modern causality relations actually have little to do with the Aristotelian concept of causation since the latter is very human but the former is (very properly) explicitly and deliberately impersonal. But the “emergence” and “downward causation” literature appears to attempt to rehabilitate Aristotelian ideas: George Ellis explicitly considers the “Four Causes” (“efficient, formal, material, final”) [54], saying that “*Fully understanding causation and fully explaining why complex systems are the way they are and behave the way they do requires holistic, historical, contextual, and extended views of causation across levels*”. Just so! He carefully and persuasively makes the case that a “holistic” approach is required to understand the behaviour of complex systems (including living ones). Our QGT approach is *explicitly* holistic in that we integrate the treatment of the local (“causal”) and the non-local (“acausal”) aspects. It is the way information flows through systems that we use to establish the impersonal phenomenon of *entropic purpose*.

Since Paul Nurse’s influential article in 2008 on “*Life, logic and information*” [89], information flows are now attracting intense interest. So, Kim et al. (2021) [90] assert that “*It is becoming increasingly imperative to develop rigorous, quantitative approaches to characterise what life is*” and concentrate on an “*information-theoretic perspective*”, as we also do. And Bielecki and Schmittel (2022) [91] concentrate on quantifying “*information encoded in structures*” in a way that seems to have some similarity to Parker and Jeynes’ (2020) [28] treatment of fullerenes. Ellis speaks of “synchronous causation” which clearly cannot be using modern ideas of *causation* since the required information cannot be passed instantaneously: the sort of phenomena Ellis has in view are a consequence of *non-local* effects (such as boundary conditions and the variational principles).

Recently, Philip Goff has proposed a radically emergent view of consciousness itself, calling it “Freedom from Physics”: “*as complex conscious systems … emerge, they bring into being new causal principles over and above the basic laws of physics* (2023 [92], p. 69).” We will not engage here with Goff’s thesis, beyond saying that in our view, his appreciation of the “laws of physics” is incomplete (leaving out a proper view of the thermodynamics). In our view, emergence is a redundant concept: at least as it has so far been applied, it points to gaps in the physical account. We believe that reality is unitary, not hierarchical: basic physics must always apply and if there exists some account of reality where it does not, then the “physics” in that account is faulty per se in some way.

It is the way information flows through systems that we will use to establish the impersonal phenomenon of *entropic purpose*. Sharma et al. [59] present “*assembly theory*” (AT) as a “*framework that … conceptualises objects not as point particles, but as entities defined by their possible formation histories … We introduce a measure called assembly, capturing the degree of causation required to produce a given ensemble of objects … AT provides a powerful interface between physics and biology. It discloses a new aspect of physics **emerging** at the chemical scale …*” They assert that “*because physics has no functional view of the Universe, it cannot distinguish novel functional features from random fluctuations, which means that talking about true novelty is impossible*”. Our view of “*entropic purpose*”, which also (like AT) captures the idea of “function”, is not “emergent” (unlike AT) but is nevertheless similar to AT in that it will also (probably) not be able to discriminate artificial intelligence (AI) entities from living ones, since it is well-known that Shannon information is impossible to distinguish from noise in the absence of the (necessarily metaphysical) decoding engine: as an example, Olivares et al. [93] contrast “Shannon entropy” and “Fisher Information”.

We should point out that both Assembly Theory [59] and Integrated Information Theory [85] have been heavily criticised on information-theoretic grounds. Hector Zenil et al. [94] have summarised their criticism of AT, pointing to their own (unacknowledged) previous work which has (i) proposed an unbiassed statistical test for whether or not the world can be regarded as “algorithmic” (2010) [95]; (ii) shown how to approximate Kolmogorov complexity measures computably (2014) [96]; and (iii) established the Block Decomposition Method (2018) [97]. Algorithmic complexity was additionally investigated in detail (2018) [98] as were also implications for evolution (2018) [99].

Another way of looking at “downward causation” (that is, “purpose”) is via the concept of “top-down control” as used by cognitive psychologists (see Chris Frith 2009 [100]). Intense experimental effort has been put into “selective attention” studies in which the subject is invited to *choose* what to pay attention to: “*The defining characteristics of top-down control are, in psychological terms, first, that we only respond to stimuli that are relevant to the task being performed, even if they are not the most salient; second, that this is a voluntary process that requires mental effort to be maintained. If our concentration lapses, we will make mistakes and respond to the wrong stimulus*.” There is copious experimental evidence for the existence of “top-down control” and therefore no doubt that “purpose” (in at least some sense) is physical.

“Downward causation” is also presented by Denis Noble [75] simply as the non-local effect of system constraints: to use differential equations for modelling the system, one must specify appropriate boundary conditions and initial conditions. Perunov et al. (2016) [101] have recently shown that many abiotic systems “*are capable of exhibiting self-organisation phenomena in the presence of dissipative external drives*” and have shown how a proper treatment of *entropy production* enables the analysis of such systems.

Philip Ball has described “*How Life Works*” [67], carefully explaining just why the “central dogma” of molecular biology (that information passes from DNA to the organism, but not from the organism to DNA) is seriously misleading: DNA should be viewed, not as a “blueprint” for life but as another organ of the cell that is carefully regulated by the organism. He quotes (*ibid*. p.83) Francesca Bellazzi [102]: “*the gene has its proper home in the cell and cannot be understood without it*”. He points out that the famous Human Genome Project resulted in a less well-known follow-up project, ENCODE (“*the Encyclopedia of DNA Elements*”) whose goal “*is to build a comprehensive parts list of functional elements in the human genome*” but has provoked bitter criticism, for example by Graur et al. [103]: “*We urge biologists not to be afraid of junk DNA … ENCODE’s take-home message that everything has a function implies purpose, and purpose is the only thing that evolution cannot provide*”, a criticism that Ball calls “*bizarre and misdirected … evolution does not pronounce the final word on what can and can’t be “functional” in biology*” (*ibid*. p.123f). ENCODE has found that most of what was considered “junk” DNA is actually transcribed, largely for functional purposes: Ball points out that for “*the individual organism, not all that is useful is heritable*” (*ibid*. p.124).

Potter and Mitchell [69] insist (citing Stuart Kauffman) that “*the true essence of the [living] system exists in the relations between [its] parts*”. This is reminiscent of Carlo Rovelli’s “relational quantum mechanics” [104] and much recent work (including Karen Barad’s insistence that the “*primary ontological unit is the phenomenon*” [70] p. 333). Holistic integration is central.

### Appendix C.6. Time and Chirality

Parker and Jeynes 2023 [3] complexify the treatment of Parker and Jeynes 2019 [13], including the complexification of time itself; a weird idea, even if it is necessary in the formalism: we have always thought of time as a “simple” scalar (even if relativity has played havoc with a naïve understanding). We could ask, with Augustine of Hippo, “*What is time?*”—but then we should take note of his famous answer: “*I know well enough what it is, provided nobody asks me*” [*quid est ergo tempus? si nemo ex me quaerat, scio*] (*Confessions* XI:14) [105].

Time has always been very puzzling even if it is supposed to be “simple”: again, Augustine understood how baffling simplicity can be when he spoke of God’s “*simple multiplicity and multiple simplicity*” [*simplici multiplicitate uel multiplici simplicitate*] (*de Trinitate* VI:6) [106]. It is interesting that at the end of a long, brilliant, and very subtle argument, Augustine ties *time* itself (in the form of recursive “remembering”) to the nature of God: “*here we are then with the mind remembering itself, understanding itself, loving itself. If we see this we see a trinity, not yet God of course, but already the image of God*” [*Ecce ergo mens meminit sui, intellegit se, diligit se. Hoc si cernimus, cernimus trinitatem, nondum quidem deum sed iam imaginem dei*] (*de Trinitate* XIV:11 [106]). It is hardly surprising that a serious treatment of time is more complicated than might have been expected!

Instead of the spatial description of stable things we have presented previously, we project them here onto the complex time plane and investigate their properties. The double-helix is the simplest stable QGT entity, being a fundamental eigenfunction of the entropic Hamiltonian. Its image in the complex time plane enables us to set up the formalism for defining “entropic purpose” as a line integral of the “purposive Lagrangian” across the complex time plane. It is essential to complexify time in order to provide a unified basis for the study of reversible and irreversible processes (see [3]). However, once a complex time plane is supposed, then all the mathematical power of complex analysis along with the remarkable properties of holomorphic functions (which are necessarily complex) become available to us.

The double-helix is a special case of the double logarithmic spiral, which is also a fundamental eigenfunction of the QGT entropic Hamiltonian. But whereas the double-helix has zero entropy production, the double logarithmic spiral has positive (non-zero) entropy production. In both cases, the entropic purpose is zero because they are both geometric (spatial) structures which have no teleological behaviour in time (as per the implicit assumptions of Shannon’s information): that is, entities with such structures are not alive per se.

It is curious that Skilling and Knuth (2019) [107] derive (reversible) quantum mechanics from symmetry and other simple basic ideas, but that they only treat pair-wise “probe/target” interactions (as complex numbers), thereby building timelessness into their formalism (automatically excluding irreversibility, and thereby automatically excluding the possibility for entropy production and therefore any allowance for entropic purpose). We also use complex numbers systematically, but explicitly in (complex) spacetime.

It is now well understood that a serious issue in Origin of Life studies is the question of the origin of the observed homochirality, given that abiotic chemistry is naturally racemic. This is modelled and discussed in detail by Chen and Ma (2020) [108]. But for our purposes, chirality is a side issue, since thermodynamics is intrinsically chiral: Parker and Walker (2010 [17]) already showed that natural DNA is expected to be right-handed, and Parker and Jeynes formalised this in Minkowski 4-space (see Appendix A of PJ19 [13]). We have emphasised the intrinsic chirality of the thermodynamical treatment by drawing Figure 2 with an unconventional axis orientation.

### Appendix C.7. Black Holes

Can black holes be said to be “alive” on the double criterion of (a) having non-zero entropy production, and (b) creating Shannon information? Clearly, criterion (a) is satisfied, but could the Hawking radiation be considered as satisfying criterion (b)? We take Kim et al.’s [90] point that “*there is no abrupt boundary between non-life and life*”, nevertheless, we would not like to propose a criterion that allowed us to think of black holes as “alive” in any sense.

Considering the current conventional wisdom, this next point must be underlined: a clear QGT treatment shows (a) that entropy production is a conserved quantity [20]; and also that (b) the entropy production of black holes has *two* components, a very small component (that corresponds to the Hawking radiation) and an enormously larger component related to the highly energetic phenomena frequently seen in the vicinity of a black hole (for example, the relativistic jets along the BH axis that characterise the active galactic nucleus often seen at the centres of galaxies inhabited by a central supermassive BH, see discussion of BHs in [20]). Therefore, it seems perverse to treat the Hawking radiation as a source of Shannon information that cannot be distinguished impersonally from noise in general since, as Alicia Juarrero says [109] (and Parker and Walker 2014 [26] prove from a different point of view): “*a communications system at thermodynamic equilibrium can transmit no actual information*”.

Perhaps adding a third criterion for life would be useful to avoid ambiguities: *autopoiesis* has a long history in the project to “define life” and has been reviewed and reformulated helpfully by Pablo Razeto-Barry [110]. “Autopoiesis” is a resonant word, recalling the Biblical account of creation: “ἐν ἀρχῇ ἐποίησεν ὁ θεὸς τὸν οὐρανὸν καὶ τὴν γῆν” [*en archē epoiēsen o theos ton ouranon kai tēn gēn*]: “*in the beginning god made the heavens and the earth*” (Genesis 1:1; LXX). All living things spontaneously make things of one sort or another. But black holes do not, so even if the Hawking radiation is accepted as exhibiting the generation of “information” (incorrectly in our view), the black hole cannot demonstrate autopoiesis. So, it is not alive. It is interesting that Razeto-Barry acknowledges that “*the autopoietic property does not explain other properties of living beings in causal terms*”, that is, the autopoietic criterion is a purely descriptive one. But still, it is useful.

## Appendix D. Information Creation, Purpose and Consciousness

Readers may think that our account of *entropic purpose* appears to quantify conscious information processing using information theoretic means in the tradition of “Integrated Information Theory” (**IIT**), introduced in 2004 by Giulio Tononi [111] (and see the recent discussion by Lombardi and López [85]). The purpose of IIT is to find a way of quantifying the “neural correlates of consciousness”, which effort has “*become mainstream*” recently, according to Max Tegmark [112]. Similarly, Seth et al. [113] open by saying, “*Any scientific study of consciousness is based on the premise that phenomenal experience is entailed by neuronal activity in the brain*”.

However, our account is entirely independent of such previous work and indeed makes no attempt to describe “consciousness” since the information creation (entropic purpose) measure we propose is entirely impersonal. In particular, no neural properties of any sort are either invoked or entailed by our account. And we emphasise that where IIT insists that the *meaning* of the information processed is of central interest, Shannon information is defined on a syntactical basis so that the semantics (the *meaning*) of the information is entirely neglected.

Seth et al. [113] “*critically examine three proposed measures of the relevant complexity of conscious neural systems: neural complexity; information integration; and a new measure, causal density*”. Their purpose is to get some sort of experimental handle on what our consciousness entails (clearly, this is related to our brains). Our intention here is to show how a truncated form of purpose (entropic purpose) may in principle be expressed physically. It is revealing that Seth et al. discuss causal density (in the context of statistical measures of Granger causality): our treatment approaches causality more generally, in a context that gives proper weight to both causality and acausality; to both local and non-local influences.

Tegmark [112] underlines the necessity of a holistic approach, saying that while *“integration as a necessary condition for consciousness is rather uncontroversial, IIT goes further and makes the bold and controversial claim that it is also a* **sufficient** *condition for consciousness, using an elaborate mathematical integration definition*” (emphases original). In that work, Tegmark was interested in computationally feasible measures of integration: here, we are interested in a more general metric for a sort of purpose (entropic purpose) that is shorn of human connotations and attributes (and in particular, independent of neuroscience) yet still depends on the creation of such information.

Peter Verheyen goes even further, making an attempt “*to describe the universe in terms of information with its biochemical/neural interpretation into a world where reality is an illusion created by the brain*” [114]. This is as it may be: our present work has no such great aspiration.

Hirsh et al. [115] have tried to use entropic methods to address “Psychological Anxiety” in an interesting and persuasive work that includes an extensive review. In an attempt to express the agency of consciousness in physical terms, they propose an “*Entropy Model of Uncertainty*”: “*individuals are motivated to keep uncertainty at a manageable level … uncertainty is experienced subjectively as anxiety*”.
